# Hypoxic tumor exosomes suppress macrophage inflammation and ferroptosis via NDUFV2 to enhance bystander tumor radioresistance

**DOI:** 10.1038/s41419-025-08357-7

**Published:** 2025-12-19

**Authors:** Jialing Zhang, Xiaoya Jin, Maidina Abulaihaiti, Xinglong Liu, Liang Zeng, Yuqi Xiao, Yan Pan, Yang Bai, Yanwu Xu, Chunlin Shao, Jianghong Zhang

**Affiliations:** 1 Institute of Radiation Medicine, Shanghai Medical College, Fudan University, Shanghai, China; 2 Radiotherapy Physics and Technology Center, Cancer Center, West China Hospital, Sichuan University, Chengdu, China; 3 Department of Biochemistry, School of Integrative Medicine, Shanghai University of Traditional Chinese Medicine, Shanghai, China; 4 Department of Radiation Oncology, Shanghai Proton and Heavy Ion Center, Fudan University Cancer Hospital, Shanghai, China

**Keywords:** Non-small-cell lung cancer, Mechanisms of disease

## Abstract

Hypoxia-induced radioresistance presents a major barrier to effective radiotherapy of non-small cell lung cancer (NSCLC), while the underlying role of macrophages remaining elusive. In this study, we found that, compared with normoxia-derived tumor exosomes, hypoxia-derived tumor exosomes more potently promoted M2 polarization of macrophages and TGF-β1/IL-10 secretion via NADH: ubiquinone oxidoreductase core subunit V2 (NDUFV2), as evidenced by the increased mitochondrial functions, including oxidative phosphorylation, ATP levels and mitochondrial membrane potentials, which collectively suppressed macrophage ferroptosis in an NDUFV2-dependent manner. When co-cultured with these exosome-educated macrophages, bystander normoxic NSCLC cells acquired radioresistance, which was attributed to the inhibition of ferroptosis. Notably, NDUFV2-knockdown macrophages abrogated this exosome-induced radioresistance. Mechanistically, IL-10 might be a pivotal signaling messenger rendering radioresistance of bystander normoxic cells. In vivo, hypoxic exosomes also conferred radioresistance to NSCLC xenografts in mice with intact macrophage populations but not in those with macrophage depletion. Immunohistochemical analysis revealed that irradiated xenografts treated with hypoxic exosomes exhibited significantly higher expression levels of GPX4, Ki67, HIF1α, CD163, and NDUFV2. Furthermore, GPX4 knockdown in xenografts reversed the radioresistance induced by hypoxic exosomes and was associated with reduced infiltration of M2-polarized macrophages, supporting the central role of ferroptosis in macrophage-regulated, hypoxia-induced radioresistance. Clinical tissue microarray analysis further confirmed that NDUFV2 was significantly upregulated in NSCLC tissues and correlated with advanced stages. Collectively, this study disclosed for the first time that hypoxic exosomes suppressed macrophage inflammatory responses and ferroptosis via NDUFV2, thereby enhancing radioresistance in bystander normoxic NSCLC cells.

## Introduction

Lung cancer remains a leading cause of cancer-related mortality globally, with non-small cell lung cancer (NSCLC) subtype accounting for approximately 85% of all lung cancers. Despite significant advances in NSCLC treatment over the past two decades [1], local recurrence following radiotherapy continues to be a major challenge due to tumor radioresistance [2]. Hypoxia, as a key hallmark of the tumor microenvironment (TME), is closely linked to tumor tolerance to radiotherapy [3], and exosomes have emerged as critical mediators of hypoxic effects in tumors [4]. Specifically, hypoxia stimulates tumor cells to release exosomes enriched with bioactive molecules and coordinates the phenotypic remodeling of innate and adaptive immune cells, enabling tumor immune evasion and promoting malignant progression. Exosomes derived from hypoxic tumor cells exert profound effects on immune cells within the TME, particularly macrophages, the most abundant infiltrating immune-associated stromal cells within and around TME [5, 6]. Tumor-associated macrophages (TAMs) exhibit distinct characteristics and metabolic profiles: M1-polarized macrophages with pro-inflammatory characteristics relying on glycolysis to fulfill their high energy demands, while M2-polarized macrophages with anti-inflammatory characteristics utilizing mitochondrial oxidative phosphorylation (OXPHOS) as their primary energy source [7–10]. The OXPHOS system comprises five multi-subunit complexes (CI-CV) encoded by the nuclear DNA and mitochondrial DNA [11]. Among these, NADH: Ubiquinone Oxidoreductase Core Subunit V2 (NDUFV2), a core subunit of the mitochondrial respiratory chain NADH dehydrogenase (CI), has been implicated in regulating mitochondrial function in adipose tissues and metabolic syndrome [12]. However, the function of NDUFV2 in macrophage polarization remains largely unclear. In addition, as an essential trace element, iron also modulates macrophage function, particularly cell development and differentiation. Iron-overloaded macrophages tend to adopt an M1 polarization through glycolysis [13, 14], whereas M2 polarization is promoted by strategies that reduce iron overload, such as iron chelators, iron inhibitors, iron-restricted diet, or hepcidin reduction [15].

Tumor radioresistance is influenced by multiple mechanisms, including the presence of cancer stem cells [16], activation of radioresistance-associated signaling pathways [17], and intercellular communication between tumor cells and immune cells [18]. Among these, ferroptosis, an iron-dependent form of cell death driven by lipid peroxidation accumulation [19], has emerged as a critical determinant of tumor radiosensitivity. Radiation can induce ferroptosis by inactivating negative regulators of this pathway, such as glutathione peroxidase 4 (GPX4) and solute carrier family 7 member 11 (SLC7A11), thereby triggering ferroptosis in tumor cells [20]. Inactivation of GPX4, together with iron overload and the peroxidation of membrane phospholipids, is associated with radiotherapy-induced cell death and tumor suppression [21]. Conversely, irradiation can also provoke an adaptive response in tumors, characterized by the upregulation of SLC7A11 or GPX4, which confers radiation tolerance [20]. The imbalance between iron uptake and export is a key factor contributing to ferroptosis dysregulation [22]. Emerging evidence highlights the crosstalk between ferroptosis and immune responses in the TME [23], with macrophages representing critical modulators of this interplay [24]. Yet, how hypoxia-induced exosomes regulates NDUFV2-mediated macrophage ferroptosis and tumor radioresistance remains undefined.

In this study, we mimicked the development of solid tumors under distinct oxygen tensions by two complementary approaches: incubating macrophages with exosomes alone, and co-culturing exosomes, macrophages, and tumor cells together. It was found that compared with normoxia-derived tumor exosomes, exosomes derived from hypoxic tumor cells markedly increased OXPHOS levels and inhibited ferroptosis in macrophages by upregulating NDUFV2 protein expression, thereby conferring immunosuppressive properties in TME. Moreover, macrophages within TME could transmit hypoxia-induced radioresistance signals to bystander normoxic tumor cells. This signal transmission further suppressed the ferroptosis pathway in the recipient normoxic tumor cells, ultimately rendering them radioresistant.

## Materials and methods

### Cell culture and treatment

Human NSCLC of A549 (wild-type p53, wtp53) and H1299 cells (p53 null) and human monocytic leukemia THP-1 cells were purchased from Cell Bank of Chinese Academy of Science (Shanghai, China) and cultured in RPMI-1640 medium (Gibco, Grand Island, NY, USA) with 10% fetal bovine serum (Yeason, Shanghai, China) and 1% penicillin/streptomycin (Gibco) at 37 °C with 5% CO_2_ in a humidified incubator (Thermofisher, Waltham, MA, USA). All cell lines were authenticated using STR profiling (Chinese Academy of Science, Shanghai, China).

### Construction of knockout and knockdown cell lines

A549 cells were electroporated with sgRNA-Cas9 complexes targeting multiple sites of the *Rab27a* gene (Gene Knockout Kit V2, Synthego™) or its negative control (MaxCyte™ GT flow transfection system). Rab27a regulates the fusion of multi­vesicular endosome (MVE) and plasma membrane and plays a critical role in exosome secretion [25]. *Rab27a*-edited (Rab27a^ko^) and control electroporated cells (1 × 10^6^ cells/ml) were expanded for 13–15 days, then Rab27a expression and knockout efficiency were assessed by Western blot.

PMA pre-stimulated THP-1 cells were transfected with *NDUFV2* siRNA (siNDUFV2) and a scrambled siRNA as control (siNC) using RFect (Bio-generating Biotechnology Co., LTD, Changzhou, China). After 48 h of transfection, cells were harvested for further application. A549 cells were infected with *GPX4* shRNA or NC-shRNA lentiviral particles for 24 h according to the manufacturer’s instruction (Hanbio Biotechnology Co., LTD, Shanghai, China). Blasticidin with the final concentration of 1 µg/ml was added to the screening medium. When A549 cells could be stably subcultured in medium containing 3 µg/ml blasticidin, the standard medium was replaced and cultured for another week. Then, Western blot was carried out to identify the knockdown efficiency of GPX4 shRNA, and the successful transfected cells were identified for subsequent experiments. The sequences of siRNA, shRNA, and guide RNAs are listed in Table 1.

**Table 1:** Oligonucleotides information.

Gene ID	Name	Sequence	Application
60	human β-actin-F	ACTCTTCCAGCCTTCCTTCC	qRT-PCR
60	human β-actin-R	CGTACAGGTCTTTGCGGATG	qRT-PCR
383	hArg1-F	TCATCTGGGTGGATGCTCACAC	qRT-PCR
383	hArg1-R	GAGAATCCTGGCACATCGGGAA	qRT-PCR
4360	CD206-F	GTGATGGGACCCCTGTAACG	qRT-PCR
4360	CD206-R	CTGCCCAGTACCCATCCTTG	qRT-PCR
4863	iNOS-F	CGTGGAGACGGGAAAGAAGT	qRT-PCR
4863	iNOS-R	CCTGGGTCCTCTGGTCAAAC	qRT-PCR
4729	hNDUFV2-F	GCACCAAUGGUUCAAAUAAAUTT	siRNA
4729	hNDUFV2-R	AUUUAUUUGAACCAUUGGUGCTT	siRNA
2879	shGPX4	Target: GTGGATGAAGATCCAACCCAA	shRNA
5873	Rab27a sgRNA-F	CCATGTGATTTGAAAACCGAGG	CRISPR KO
5873	Rab27a sgRNA-R	ATAAAGTGCTGCCTTGCACCTGG	CRISPR KO

### Hypoxia and drug treatments

A549 and H1299 cells were maintained in a hypoxia workstation (Don Whitley Scientific, UK) with a humidified and stable mixture of 1% O_2_, 5% CO_2_, and 94% N_2_ at 37 °C for 48 h and then their media were collected for exosomes isolation under a hypoxic environment. As previously reported, GW4869 is a common inhibitor of exosome biogenesis/release [26]. To inhibit exosome release under hypoxia treatment, A549 and H1299 cells were administrated with 10 mM GW4869 (MedChemExpress, Monmouth Junction, NJ, USA) for 48 h before harvesting the supernatant under hypoxic condition. For inhibition of IL-10/IL-10RA downstream signaling, A549 cells were treated with 20 nM Ruxolitinib (a selective JAK1/2 inhibitor, Selleck) for 24 h under normoxic condition prior to irradiation.

THP-1 cells (1 × 10^6^) were plated in 6-well plates and cultured in a fresh complete medium containing 100 ng/ml of phorbol 12-myristate 13-acetate (PMA, MedChemExpress) for 24 h to induce M0 macrophage differentiation. Morphological observation and CD11b expression detection were performed to validate cell differentiation into macrophages.

### Isolation and identification of exosomes

Cells were cultured to 50% confluence and washed with phosphate-buffered saline (PBS). Then the medium was replaced with RPMI-1640 containing 10% exosome-depleted FBS (Absin, Shanghai, China). After culturing under normoxic (21% O_2_) or hypoxic (1% O_2_) condition for 48 h, cell culture supernatants were collected for exosome isolation as previously described [27]. Exosomes were categorized as O_2_-exo (from normoxic cells), N_2_-exo (from hypoxic cells), N_2_-Rab27a^ko^-exo (from hypoxic Rab27a^ko^ cells), and N_2_-GW-exo (from hypoxic cells pre-treated with GW4869, an exosome secretion inhibitor). A549-Rab27a^ko^ cells were employed to generate N_2_-Rab27a^ko^-exo as a control for N_2_-exo.

To identify the hallmarks of exosomes, the isolated exosomes were observed using transmission electron microscopy and detected with a nanoparticle tracking analysis (ZetaVIEW version 8.05.14) (SP7, Particle Metrix, Meerbusch, Germany) based on the reported protocols [28].

### Exosome dosage

Under different cell culture conditions, exosome pellets were collected from the same amount of tumor cells (8 × 10^6^ cells) and suspended in the same amount of PBS (200 μl). Exosomal protein concentrations were determined using the BCA protein quantification kit (Thermo Fisher Scientific Inc., Waltham, MA) according to the manufacturer’s instructions. Exosome amounts were standardized across groups based on protein concentration and source cell number. For in vitro experiments, macrophages were co-cultured with 25 μl exosome suspension per ml of medium supplemented with exosome-depleted FBS, corresponding to ~5 μg exosomal protein per well in 6-well plates. For in vivo experiments, mice received peritumoral injections of 20 μl exosome suspension (~1.2 μg protein) every other day.

### Exosome uptake

To demonstrate the uptake of exosomes by THP-1 cells, THP-1 cells were pre-stained with 10 μM of Dio (Beyotime, Shanghai, China). Then, the exosomes were labeled with PKH26 (Umibio, Shanghai, China) according to the manufacturer’s instructions. Briefly, exosomes were incubated with 100 μM PKH26 for 10 min in the dark. The excess PKH26 dye was removed by ultracentrifugation at 35,000 rpm for 4 h at 4 °C (Beckman-Coulter OptimaXE-100/XE-90, Brea, CA, USA). The exosome pellets were washed triply with PBS. The pre-stained THP-1 cells were co-cultured with PKH26-labeled exosomes for 0, 2, 4, or 6 h and fixed with 4% paraformaldehyde, then the cell nuclei were stained with DAPI solution (Beyotime). Exosome uptake was observed and photographed using a laser confocal microscope (Leica, SP8, Heidelberg, Germany).

### Colony formation assay

The radiosensitivities of NSCLC cell lines co-cultured with or without THP-1 cells and exosomes were determined using a colony formation assay. Tumor cells were detached from the lower chamber and immediately irradiated with 6 Gy of X-rays at a dose rate of 1 Gy/min (X-RAD 320, Precision X-ray, Inc., North Branford, CT, USA; 12 mA, 2-mm aluminum filtration), followed by trypsin treatment to generate a single cell suspension and plated in triplicate in 6-well plates at suitable density. The cells were cultured for 2 weeks and fixed with methanol for 20 min. After staining with crystal violet (Beyotime), the visible colonies containing at least 50 cells were counted.

### Flow cytometry analysis

The Fc receptors of THP-1 cells were blocked at room temperature for 10 min by Human TruStain FcX^TM^ (BioLegend, San Diego, CA, USA). Macrophages were incubated with anti-human fluorescent antibodies in the dark at 4 °C for 30 min, including CD11b (FITC), CD86 (PE), and CD163 (PE/Cy7), then washed with cell stain buffer (4abio, Suzhou, China). Singlet collected from xenograft tumors (see below) was suspended in PBS. To confirm the living part of the cells in singlet, cells mixed with Fixable Viability Stain 780 (BD Pharmingen, San Diego, CA, USA) were incubated at room temperature for 10 min and washed with cell stain buffer. Next, the Fc receptors were blocked with anti-mouse TruStain FcX™ PLUS (BioLegend) at room temperature for 10 min, and then the cells were stained with anti-mouse fluorescent antibodies in the dark at 4 °C for 30 mins, including CD45 (PerCP/Cy5.5), F4/80 (FITC), CD11b (PE/Cy7), CD86 (PE) and CD163 (APC) (BioLegend). The expression levels of surface markers were analyzed using a CytoFLEX cytometer (Beckman-Coulter, USA).

### Quantitative real-time PCR assay

Total cellular RNA was extracted from THP-1 cells using TRIzol reagent (Invitrogen, San Diego, CA, USA) and reversely transcribed into cDNA using ABScript III RT Master Mix for qPCR with gDNA Remover (ABclonal, Wuhan, China). qRT-PCR was performed in 20 μl reaction reagent using SuperReal PreMix Plus (SYBR Green) (Tiangen, Beijing, China) on the CFX Opus 96 platform (Bio-Rad, Hercules, CA, USA). The relative quantification of gene expression was assessed by comparative threshold cycle (Ct), and the fold change of target genes was calculated in a 2^−ΔΔCt^ manner. The specific primers of the target genes (human: Arg1, CD206, iNOS, and NDUFV2) and the reference gene (β-actin) are listed in Table 1.

### ELISA assay

THP-1 cells were treated with exosomes for 24 h, and the supernatants were collected and centrifuged at 1000 × *g* for 10 min. Subsequently, the cytokines of TGF-β1 and IL-10 in the supernatants or medium containing naïve exosomes were measured using corresponding ELISA Kits (RK00055, RK00012, ABclonal). TGF-β1 and IL-10 in the mice serum at 24 days post IR were measured using corresponding ELISA Kits (RK00057, RK00016, ABclonal). The OD values were detected at a wavelength of 450 nm, and the cytokine concentration was calculated according to the standard curve.

### Measurement of oxygen consumption rate

THP-1 cells were seeded in XF96-cell culture plates (Agilent, Santa Clara, CA, USA) with a density of 1 × 10^4^ cells/well. After being treated with exosomes for 24 h, the medium was replaced with 180 μl RPMI-1640 containing 1 mM pyruvate, 2 mM glutamine, and 10 mM glucose. According to the manufacturer’s instructions, XF-96 plates were equilibrated in a non-CO_2_ incubator at 37 °C. The metabolic toxins containing ATP synthase inhibitor oligomycin (0.5 μM), mitochondrial OXPHOS uncoupling agent FCCP (1 μM), mitochondrial complex I inhibitor rotenone (0.5 μM), and mitochondrial complex III inhibitor antimycin A (1.5 μM) were put into the cartridge drug ports. The cellular oxygen consumption rate (OCR) was measured in real time by loading the drugs in order using a Seahorse XFe96 analyzer (Agilent). Data were analyzed by Seahorse Wave Controller (v2.6.3, Agilent). The basal respiration, ATP production, proton leak, maximal respiration, and spare capacity were calculated according to previous reports [29, 30].

### Intracellular ATP assay

Intracellular ATP level of THP-1 cells was determined using an enhanced ATP Assay Kit (Beyotime). Briefly, after 24 h of treatment, the medium was discarded, then 200 μl of lysate per well was added for full lysis at 4 °C, followed by centrifugation at 12,000 × *g* for 5 min at 4 °C. The supernatant was collected to measure the intracellular ATP level using a microplate reader, and the protein concentration was detected using the BCA protein assay kit (Beyotime) for normalization.

### JC-10 staining

THP-1 cells were plated in triplicate in 24-well plates and treated with exosomes for 6 h. Mitochondrial membrane potential (MMP, ΔΨm) was assessed by JC-10 (4abio) according to the manufacturer’s protocols. The increase in ΔΨm was evaluated by the transition from aggregates (red fluorescence) to monomer (green fluorescence). The degree of mitochondrial functional integrity and possible ferroptosis inhibition was evaluated regarding the red/green fluorescence intensity ratio.

### Phagocytosis assay

THP-1 cells were incubated with the pre-warmed medium containing Latex Beads-Rabbit IgG-PE Complex (600541, Cayman, USA) at a dilution of 1%. Then, the cells were washed with PBS and fixed with 4% formaldehyde (Servicebio, Wuhan, China) for 15 min. The cell nuclei were stained with 1.43 µM DAPI (Beyotime). The uptake of IgG-PE beads engulfed by macrophages was observed using a fluorescence microscopy, and corresponding images were photographed and analyzed using the Image J software.

### Western blot assay

Total cellular protein extraction was performed using RIPA buffer containing 100 mM phenylmethanesulfonyl fluoride (PMSF, Beyotime) with a loading buffer (Beyotime) containing protease inhibitors. An equal amount of protein was subjected to 10% SDS-PAGE gel at a constant voltage and then transferred to a polyvinylidene difluoride (PVDF) membrane (0.45 µm, Millipore, Burlington, MA, USA) for further antibody binding reactions. After being blocked with 5% nonfat milk in Tris-buffered saline/Tween 0.05% (TBST) for 1.5 h, the PVDF membranes were incubated with primary antibodies (Table 2) at 4 °C overnight. After incubated with suitable secondary antibodies (1:3000, Beyotime) for 1.5 h, the proteins were finally detected using an ECL kit (Tanon, Shanghai, China) and analyzed using the ChemiDoc XRS system (Tanon).

**Table 2:** Antibodies and recombinant protein information.

Name	Supplier	Cat. no.	Application
Rab27a rabbit pAb	ABclonal	A1934	WB (1:1000)
FTL rabbit mAb	ABclonal	A11241	WB (1:1000)
FTH1 rabbit mAb	ABclonal	A19544	WB (1:1000)
TfR1 rabbit pAb	ABclonal	A18083	WB (1:1000)
FPN rabbit pAb	ABclonal	A14884	WB (1:1000)
PGC1α rabbit pAb	ABclonal	A12348	WB (1:1000)
SDHA rabbit mAb	ABclonal	A13852	WB (1:1000)
UQCRC2 rabbit pAb	ABclonal	A4181	WB (1:1000)
COX15 rabbit pAb	Proteintech	11441-1-AP	WB (1:1000)
TFB2M rabbit pAb	Proteintech	24411-1-AP	WB (1:1000)
TFAM rabbit pAb	Proteintech	22586-1-AP	WB (1:1000)
ATP5H rabbit pAb	Proteintech	17589-1-AP	WB (1:1000)
CD81 rabbit pAb	Proteintech	27855-1-AP	WB (1:1000)
pSTAT3(Tyr705) rabbit mAb	Abmart	T56566	WB (1:1000)
STAT3 rabbit mAb	Abmart	T55292	WB (1:1000)
β-tubulin rabbit mAb	ABclonal	A12289	WB (1:2000)
β-actin rabbit mAb	Beyotime	AF5003	WB (1:2000)
CD11c rAb	Abcam	ab219799	IHC (1:500)
CD19 rAb	Abcam	ab245235	IHC (1:1000)
CA9 rAb	Proteintech	84233-1-RR	IHC (1:500)
CD163 rabbit pAb	Abcam	ab182422	IHC (1:500)
GPX4 rabbit mAb	Abcam	ab125066	WB(1:1000) IHC (1:200)
NDUFV2 rabbit mAb	Abcam	ab183715	WB(1:1000) IHC (1:500)
Ki67 rabbit pAb	Servicebio	GB111499-100	IHC (1:500)
HIF1α rabbit pAb	Proteintech	20960-1-AP	IHC (1:200)

### Detection of intracellular ROS

Cells were seeded in 6-well plates at a density of 2 × 10^5^ per well and subjected to the indicated treatments. After incubation or irradiation, the supernatants were removed and cells were washed with PBS. Cells were then incubated with 2 μM 2′,7′-dichlorofluorescein diacetate dye probe (DCFH-DA, Beyotime) at 37 °C in a humidified 5% CO_2_ incubator for 30 min, followed by washing with PBS to remove excess probe. Fluorescence intensity was analyzed using a CytoFLEX flow cytometer (Beckman-Coulter), with 10000 cells collected per sample.

### Measurement of GSH/GSSG

Cells were seeded in 6-well plates at a density of 2 × 10^5^ cells per well, and after collection cell supernatants were prepared for measurement of GSH/GSSG using the GSH and GSSG Assay Kit (Beyotime) according to the manufacturer’s instructions. The concentrations of total glutathione (GSH + GSSG) and oxidized glutathione (GSSG) were determined by measuring absorbance at 405 nm with a microplate reader. The concentration of reduced glutathione (GSH) was calculated as the total glutathione minus twice GSSG.

### Detection of intracellular irons

Intracellular Fe^2+^ was detected using FerroOrange (Dojindo). Briefly, after 6 h treatment of exosomes, THP-1 cells were incubated with 1 μM FerroOrange in Hank’s balanced salt solution (HBSS, Beyotime) for 30 min at 37 °C. Fluorescence images were photographed with an ImageXpress Micro 4 screening system (Molecular Devices, San Jose, CA, USA). Mean fluorescence intensity (MFI) was analyzed using Image J software (v2.14.0, National Institutes of Health, USA).

### Lipid peroxidation assay

Intracellular lipid peroxidation accumulation was detected by C11/BODIPY fluorescence probe (Abclonal). Briefly, the suitably treated cells were incubated with 5 μM C11/BODIPY fluorescence probe at 37 °C in the dark for 30 min. The intracellular lipid peroxidation levels were assessed by the ratio of oxidized form of C11/BODIPY to reduced form of C11/BODIPY using a high-content imaging system. MFI was analyzed with the ImageJ software.

### Co-culture of macrophages and NSCLC cells

Activated THP-1 cells (5 × 10^5^) were treated with exosomes or PBS and plated on the insert chamber with a pore size of 0.4-μm (#3450, Corning, NY, USA), and A549 cells (2 × 10^5^) were seeded on the lower chamber of the 6-well plate. These cells were co-cultured for 24 h, then the A549 cells were detached for subsequent experiments.

### Animal experiments

Five-week-old BALB/c nude male mice (SPF Biotechnology CO., Ltd, Beijing, China) were maintained at a standard condition (24 °C temperature, 50% relative humidity, and 12 h light/dark cycle) for 1 week before experiments. For macrophage depletion, 200 μl of clodronate liposomes (5 mg/ml, LIPOSOMA, Netherlands) were intraperitoneally injected two days before tumor-bearing, then 100 μl of clodronate liposomes (abbreviated as Clo below) was maintained every 4 days. The macrophage clearance efficiency was validated by flow cytometry assay. To establish tumor xenograft model, we injected A549 cells and A549 cells transfected with GPX4 shRNA or its negative control cells (5 × 10^6^/100 μl) subcutaneously into the right limb of mice.

When the tumor size reached about 100 mm^3^, the nude mice were randomly divided into groups and peritumorally injected with 20 μl of PBS, O_2_-exo, N_2_-exo, or N_2_-Rab27a^ko^-exo, once every other day until the mice were sacrificed. All exosomes were derived from A549 cells and filtered using 0.22 μm sterile filters before injection. On the day after the first exosome administration, xenografts were treated with local IR of 20 Gy X-ray. Thus, there were ten A549 xenograft groups in total, including: nonirradiated (nonIR) mice bearing A549 cells administered with PBS, O_2_-exo, N_2_-exo, or N_2_-Rab27a^ko^-exo and nonIR macrophage-deficient mice bearing A549 cells administered with N_2_-exo, and corresponding IR groups, which were abbreviated as PBS, O_2_-exo, N_2_-exo, N_2_-exo + Clo, N_2_-Rab27a^ko^-exo, PBS + IR, O_2_-exo + IR, N_2_-exo + IR, N_2_-exo + Clo + IR and N_2_-Rab27a^ko^-exo + IR. Besides, there were another four groups of A549-NC or A549-GPX4-sh xenograft, including: nonIR mice bearing A549-NC cells administered with N_2_-exo, nonIR mice bearing A549-GPX4-sh cells administered with N_2_-exo, and corresponding IR groups, which were abbreviated as NC + N_2_-exo, GPX4-sh + N_2_-exo, NC + N_2_-exo + IR, and GPX4-sh + N_2_-exo + IR.

In IR groups, mice were anesthetized with 2.5% tribromoethanol (Sigma-Aldrich Corp, St. Louis, MO, USA) and placed in a well-ventilated lead mold, allowing local IR of xenograft with a single dose of 20 Gy X-rays at a dose rate of 2 Gy/min [27]. The tumor volumes were measured every 3 days and calculated with the equation: V = (L × W^2^) × 0.5 (L = length, W = width). Finally, the mice were sacrificed by cervical dislocation after blood collection from the eyeball under anesthesia. Then, the xenograft tumor was dissected, photographed, and subjected to subsequent flow cytometry, western blot, and immunohistochemical analysis. All animal experimental procedures and unblinded operations were approved by the Animal Welfare and Ethics Committee of Fudan University in compliance with the ARRIVE guidelines, which were carried out following the National Institutes of Health guide for the care and use of laboratory animals.

### Tissue immunohistochemical analysis

Mouse xenograft tumor samples were fixed in 4% paraformaldehyde, embedded in paraffin, and cut into 4-μm-thick tissue slices. After dewaxing, dehydration, and blocking endogenous peroxidase and nonspecific binding sites, the tissue sections were incubated with primary antibodies against HIF1α (1:200, Proteintech, Wuhan, China), Ki67 (1:500, Servicebio), GPX4 (1:200, Abcam, Cambridge, MA, USA) and CD163 (1:500, Abcam), respectively, at 4 °C overnight. Then tissue sections were washed triply with PBST and incubated with HRP-labeled secondary goat antibody (1:200, Servicebio) for 50 min at room temperature, followed by treatment with DAB (DAKO, Glostrup, Denmark) and hematoxylin staining. To further evaluate the expressions of GPX4, NDUFV2, and CD163 in tumor xenografts, we performed multiple fluorescent immunohistochemical staining using a four-color multiplex fluorescent immunohistochemical staining kit (abs50028, Absin, China) according to the manufacturer’s instructions. IHC images in at least three fields were randomly obtained at ×400 magnification using Slideview VS200 (Olympus, Tokyo, Japan) and analyzed with Image J software. The fluorescence expression levels were analyzed using AP-TIME (3DMed Diagnostics, Shanghai, China).

### Tissue microarray analysis

A human lung adenocarcinoma (LUAD) tissue microarray (Cat no. HLugA180Su12, Outdo Biotech, Shanghai, China) containing paired tumor and adjacent normal tissues was used. Sections were deparaffinized, rehydrated, subjected to antigen retrieval, and incubated with antibody against NDUFV2 (1:500, Abcam, Cambridge, MA, USA) overnight at 4 °C, followed by IHC procedures mentioned above. Digital images of stained sections were obtained by Slideview VS200 (Olympus, Tokyo, Japan) and analyzed using QuPath version 0.6.0 [31]. Four paired samples were excluded from the analysis due to insufficient tissue coverage, as their inclusion could introduce potential bias.

### Statistical analyses

The experimental data were analyzed using GraphPad Prism 10 software (San Diego, CA, USA). Results were expressed as the mean ± standard error mean for at least three independent experiments. Statistical significance was evaluated using Student’s *t* test between two groups and using one-way analysis of variance (ANOVA) for more than two groups. *P* < 0.05 was considered statistically significant.

### Ethics

The study was conducted according to the guidelines of the Declaration of Helsinki and approved by the Animal Welfare and Ethics Committee of Fudan University.

## Results

### The characteristics of exosome relied on oxygen conditions in a Rab27a-dependent manner

Cellular exosomes were isolated and enriched according to a previously published protocol [27]. The exosomes exhibited a typical morphology of round or oval membranous vesicles (Fig. S1A). Based on the dynamic changes in cell proliferation and exosomal protein content observed at 24, 48, and 72 h under hypoxic conditions, 48 h was selected as the optimal time point for exosome collection (Fig. S1B). Given the regulatory role of Rab27a in the fusion of MVEs and plasma membrane, it was found that Rab27a was significantly high-expressed in the hypoxic A549 cells in comparison with normoxic cells (Fig. S1C). To inhibit the release of exosomes, the Rab27a gene was knocked out by the CRISPR/Cas9 system with high efficiency (Fig. S1D). NTA analysis showed that the mean size of O_2_-exo was obviously larger than that of N_2_-exo, although the number of O_2_-exo was almost half of N_2_-exo from the same number of cells under different oxygen conditions, and the exosome secretion was eliminated by Rab27a knockout as well as GW4869 (Fig. S1E). Moreover, the content of protein in N_2_-exo was slightly higher than that in O_2_-exo from the same number of cells under different oxygen conditions (Fig. S1F), indicating that, due to owing larger sizes, hypoxic exosomes may contain much more protein molecules than normoxic exosomes.

### Hypoxic exosomes induced M2 polarization of macrophages

**Fig. 1:**
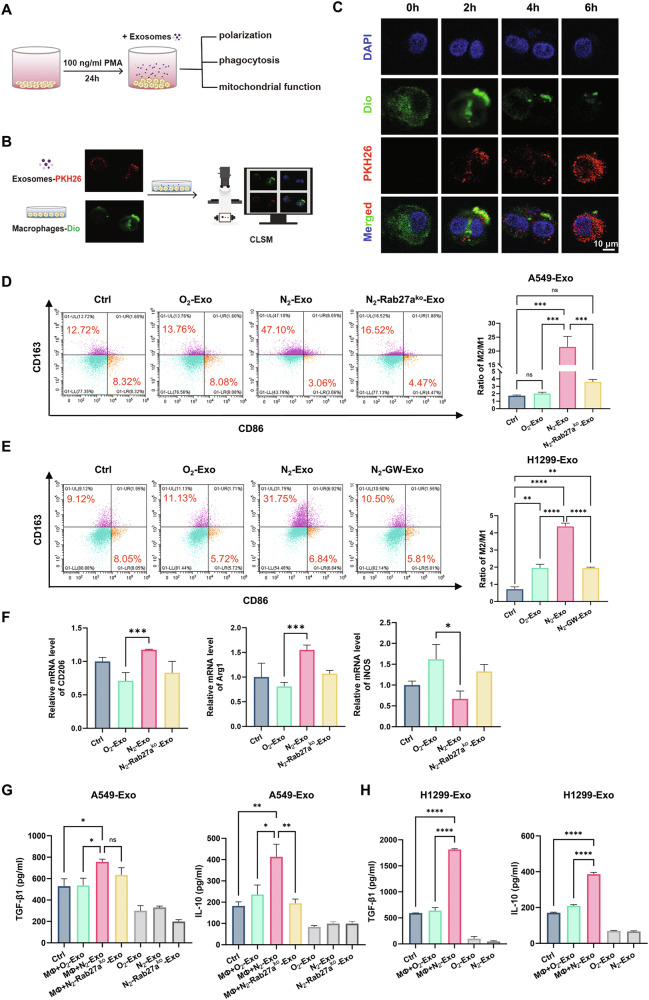
Hypoxic exosomes promoted M2 polarization of macrophages. **A** THP-1 cells were pretreated with 100 ng/ml PMA for 24 h to induce M0 macrophages, then incubated with exosomes. Subsequently, polarization, phagocytosis, and mitochondrial functions were detected. **B** THP-1 cells were pre-stained with 10 μM Dio and then co-cultured with A549 cells-derived exosomes pre-labeled with 100 μM PKH26 for confocal microscopy observation. **C** Representative fluorescence images of THP-1 cells (green) treated with A549-derived exosomes (red) and then stained with 1.43 µM DAPI (blue) for visualization of exosome uptake. Scale bar, 10 μm. Flow cytometry analysis of CD86 and CD163 in THP-1 cells after incubation with exosomes derived from A549 cells (**D**) and H1299 cells (**E**). **F** Relative mRNA expression levels of M2 markers (CD206, Arg1) and the M1 marker (iNOS) in THP-1 cells following exosome treatment, determined by qRT-PCR. ELISA analyses of TGF-β1 and IL-10 in the supernatants of THP-1 cells after 24 h incubation with exosomes from A549 cells (**G**) and H1299 cells (**H**), PBS as control of exosomes. *P < 0.05; **P < 0.01; ***P < 0.001; ****P < 0.0001; ns no significance.

To explore whether tumor exosomes regulate macrophage polarization, we incubated PMA-activated THP-1 cells with exosomes isolated from NSCLC cells under different conditions, then assessed macrophage polarization, phagocytic activity, and mitochondrial function (Fig. 1A). Overnight treatment of PMA (100 ng/ml) successfully differentiated THP-1 cells into M0 macrophages, as confirmed by the expression level of CD11b (Fig. S2A, B) and bright-field microscope observation (Fig. S2C). It was found that macrophages efficiently uptook these exosomes in a time-dependent manner (Fig. 1B, C). Moreover, the expression of CD163 (M2 marker) in macrophages was slightly increased by O_2_-exo, N_2_-Rab27a^ko^-exo, or N_2_-GW-exo, but extensively increased by N_2_-exo derived from both A549 and H1299 cells; while the expression of CD86 (M1 marker) was decreased so that the ratio of M2/M1 had a higher level in N_2_-exo group in comparison with other groups (Fig. 1D, E). Consistently, N_2_-exo significantly upregulated the mRNA expression of M2-associated genes (Arg1 and CD206) while downregulating the M1 marker gene iNOS (Fig. 1F), confirming M2 polarization at the transcriptional level. Since M2-polarized macrophages could secrete anti-inflammatory cytokines, the concentration of transforming growth factor-β1 (TGF-β1) and interleukin 10 (IL-10) in the supernatant of macrophages were significantly increased by N_2_-exo (Fig. 1G, H). As expected, inhibiting tumor exosome secretion via Rab27a knockout reversed the N_2_-exo-induced elevation of TGF-β1 and IL-10 in macrophage supernatants, with a more pronounced effect on IL-10. In addition, the exosomes themselves contained low levels of TGF-β1 and IL-10, with no significant differences among different groups, indicating that TGF-β1 and IL-10 were mainly released from macrophages. Collectively, these results demonstrated that hypoxic NSCLC cell-derived exosomes could markedly promote M2 polarization of macrophages.

### Hypoxic exosomes promoted macrophage M2 polarization and enhanced mitochondrial function via NDUFV2

**Fig. 2:**
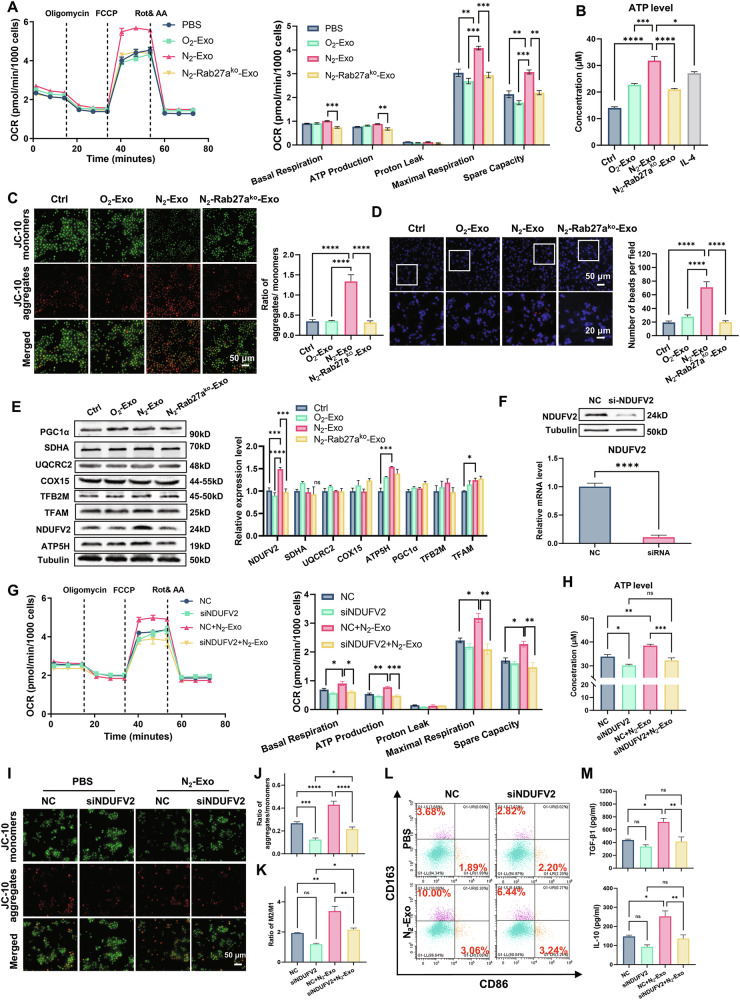
Hypoxic exosomes promoted M2 polarization and mitochondrial functions of macrophages via NDUFV2. **A** Effects of exosomes on oxidative phosphorylation (OXPHOS) of THP-1 cells detected by Seahorse assay. Key parameters of basal respiration, ATP production, proton leak, maximal respiration, and spare capacity, reflected mitochondrial functions of THP-1 cells incubated with A549 cells-derived exosomes or PBS, as determined by sequential administration of 0.5 μM oligomycin, 1 μM FCCP, and 0.5 μM rotenone and 1.5 μM antimycin A. **B** Intracellular ATP level in THP-1 cells after 24 h incubation with exosomes. **C** Representative fluorescence images and analysis of JC-10-stained THP-1 cells after 6 h of PBS or exosomes treatment. Scale bar, 50 μm. **D** Representative fluorescence images and quantification of THP-1 cells incubated with exosomes engulfing IgG-PE beads. After incubation with exosomes or PBS for 24 h, THP-1 cells were co-cultured with IgG-PE beads (red) for 1 h and then stained with 1.43 µM DAPI (blue). Scale bars, 50 μm (top) and 20 μm (bottom). **E** Western blot assay of proteins and their relative levels in THP-1 cells at 6 h after incubation with exosomes as indicated. **F** Protein and RNA expression levels of NDUFV2 in THP-1 cells transfected with siNDUFV2 and its negative control, respectively. **G** Effects of siNDUFV2 on OXPHOS of THP-1 cells treated with N_2_-exo. **H** Intracellular ATP level of THP-1 cells transfected with siNDUFV2 after 24 h incubation with N_2_-exo. **I**, **J** Representative fluorescence images and quantification of JC-10-stained THP-1 cells transfected with siNDUFV2 after 6 h of incubation with N_2_-exo. Scale bar, 50 μm. **K**, **L** Flow cytometry analysis of CD86 and CD163 in THP-1 cells transfected with siNDUFV2 after 24 h incubation with N_2_-exo. **M** ELISA analyses of TGF-β1 and IL-10 expressions in the supernatant of THP-1 cells transfected with siNDUFV2 after 24 h incubation with N_2_-exo. *P < 0.05; **P < 0.01; ***P < 0.001; ****P < 0.0001; ns no significance.

Hypoxia has been proven to alter the metabolic pathway of macrophages [10]. We hypothesized that the promotion of M2 polarization by hypoxic exosomes might also modulate mitochondrial OXPHOS. Our results showed that the OCR of macrophages was increased after incubation with N_2_-exo, as evidenced by the significant elevations in maximal respiration and spare capacity (Fig. 2A). Furthermore, the intracellular ATP levels of macrophages were also significantly increased after incubation with N_2_-exo from both A549 cells (Fig. 2B) and H1299 cells (Fig. S3A), which was consistent with the IL-4 treated group. IL-4 is a classical inducer of macrophage M2 polarization, hence its use as a positive control in this context. Moreover, the mitochondrial membrane potentials (MMPs) of macrophages were also increased by N_2_-exo, as indicated by increased JC-10 aggregate fluorescence(red) and decreased JC-10 monomer fluorescence (green) (Fig. 2C). Notably, the macrophages engulfed more IgG-PE beads after incubation with N_2_-exo from A549 cells (Fig. 2D) or H1299 cells (Fig. S3B), indicating that hypoxic exosomes facilitated macrophage phagocytosis. In contrast, O_2_-exo and N_2_-Rab27a^ko^-exo derived from the same number of tumor cells had less effects on macrophage mitochondrial function upon OXPHOS, ATP generation, MMPs, and phagocytic capacity.

To elucidate the mechanism underlying the above effects, we detected the expressions of key proteins involved in mitochondria biogenesis and OXPHOS, including NDUFV2, SDHA, UQCRC2, COX15, ATP5H, PGC1α, TFAM, and TFB2M. It was found that, among these proteins, only NDUFV2 was most significantly increased in the macrophages after N_2_-exo treatment, but it had no alterations under O_2_-exo or N_2_-Rab27a^ko^-exo treatment (Fig. 2E). We further examined the exosomal cargo and confirmed that N_2_-exo carried more NDUFV2 than O_2_-exo (Fig. S4A). Direct supplementation of recombinant NDUFV2 protein into macrophages also recapitulated the mitochondrial changes induced by N_2_-exo (Fig. S4B, C), suggesting that NDUFV2 was a functional mediator of exosome-induced effects. To further validate this, we transfected the macrophages with NDUFV2 siRNA (siNDUFV2) with a knockdown efficiency of about 90% (Fig. 2F), and then re-evaluated the effects of exosomes on macrophages. Results showed that siNDUFV2 transfection diminished the respiratory capacity of mitochondria and ATP levels in the macrophages under N_2_-exo incubation (Fig. 2G, H). JC-10 staining further revealed that NDUFV2 knockdown impaired MMPs in macrophages, and this membrane depolarization could not be reversed by N_2_-exo treatment (Fig. 2I, J). Moreover, NDUFV2 knockdown also eliminated the influence of N_2_-exo on macrophage polarization, evidenced by a reduced M2/M1 ratio (Fig. 2K, L) and decreased expressions of TGF-β1 and IL-10 (Fig. 2M). Conclusively, NDUFV2 played essential roles in N_2_-exo-promoted macrophage M2 polarization and mitochondrial functional integrity.

### Hypoxic exosomes suppressed macrophage ferroptosis via NDUFV2

**Fig. 3:**
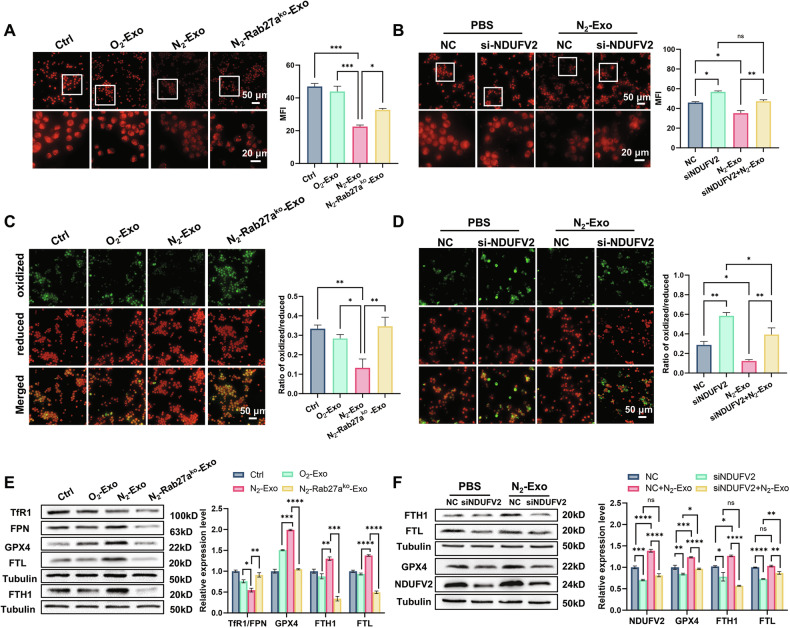
Hypoxic exosomes promoted ferroptosis inhibition of macrophages through high expression of macrophage NDUFV2. Representative fluorescence images of FerroOrange-stained THP-1 cells (**A**) and THP-1 cells transfected with siNDUFV2 (**B**) after 6 h of incubation with indicated exosomes, and the analyses of intracellular Fe^2+^ levels. Scale bar, 50 μm (top) and 20 μm (bottom). Representative fluorescence images and quantification of lipid peroxidation in THP-1 cells (**C**) and THP-1 cells transfected with siNDUFV2 (**D**) after 6 h incubation with indicated exosomes. Scale bar, 50 μm. **E** Western blot assay of TfR1, FPN, GPX4, FTH1, and FTL proteins and their relative expression levels in THP-1 cells after 6 h incubation with indicated exosomes. **F** Western blot assay of GPX4, FTH1, FTL, NDUFV2 proteins and their relative expression levels in THP-1 cells transfected with siNDUFV2 after 6 h incubation with indicated exosomes. *P < 0.05; **P < 0.01; ***P < 0.001; ****P < 0.0001; ns no significance.

MMP helps evaluate mitochondrial functional status, and abnormal MMP is regarded as an early warning of ferroptosis [32]. While iron has been shown to regulate macrophage polarization through multiple mechanisms [33]. However, the intrinsic relationship between ferroptosis and macrophage phenotype remains incompletely understood. Given our finding that hypoxic exosomes could significantly increase MMPs of macrophages via NDUFV2, we next investigated how ferroptosis status could be altered alongside NDUFV2 upregulation in response to hypoxic exosomes. Consistent with MMP elevation, N_2_-exo treatment decreased ROS level and increased GSH/GSSG ratio in macrophages, indicating the inhibitory effect of hypoxic exosomes on ferroptosis (Fig. S4D, E). We further found that the intracellular Fe^2+^ levels were reduced in macrophages after N_2_-exo incubation, while this decrease was not observed in the O_2_-exo group (Fig. 3A). Notably, N_2_-Rab27a^ko^-exo also reduced intracellular Fe^2+^ levels in macrophages, although it was not as effective as N_2_-exo. But NDUFV2 knockdown enhanced the intracellular Fe^2+^ levels in macrophages (Fig. 3B).

It’s well known that the accumulation of lipid peroxidation features ferroptosis [19]. C11-BODIPY staining revealed that N_2_-exo mitigated lipid peroxidation in macrophages (Fig. 3C), whereas NDUFV2-silenced macrophages exhibited increased accumulation of lipid peroxidation (Fig. 3D). Then, we detected the expression of ferroptosis inhibition-related proteins and iron homeostasis-related proteins in macrophages, and found that the expression of GPX4 was increased in macrophages incubated with N_2_-exo or O_2_-exo, especially N_2_-exo (Fig. 3E). Similarly, supplementation with recombinant NDUFV2 protein elevated the expression of GPX4 and FTH1 in macrophages (Fig. S4F), reinforcing NDUFV2’s role in inhibiting ferroptosis. Additionally, N_2_-exo reduced the ratio of TfR1 to FPN, a marker of intracellular iron load, while simultaneously upregulating FTH1 and FTL. Importantly, NDUFV2 knockdown abrogated N_2_-exo-induced upregulation of GPX4, FTH1, and FTL (Fig. 3F), suggesting that N_2_-exo alleviated macrophage iron overload in an NDUFV2-dependent manner. Collectively, these findings demonstrated that hypoxic tumor exosomes maintained macrophage viability by suppressing ferroptosis through NDUFV2.

### Hypoxic exosomes-educated macrophages induced radioresistance in normoxic A549 cells via ferroptosis inhibition

**Fig. 4:**
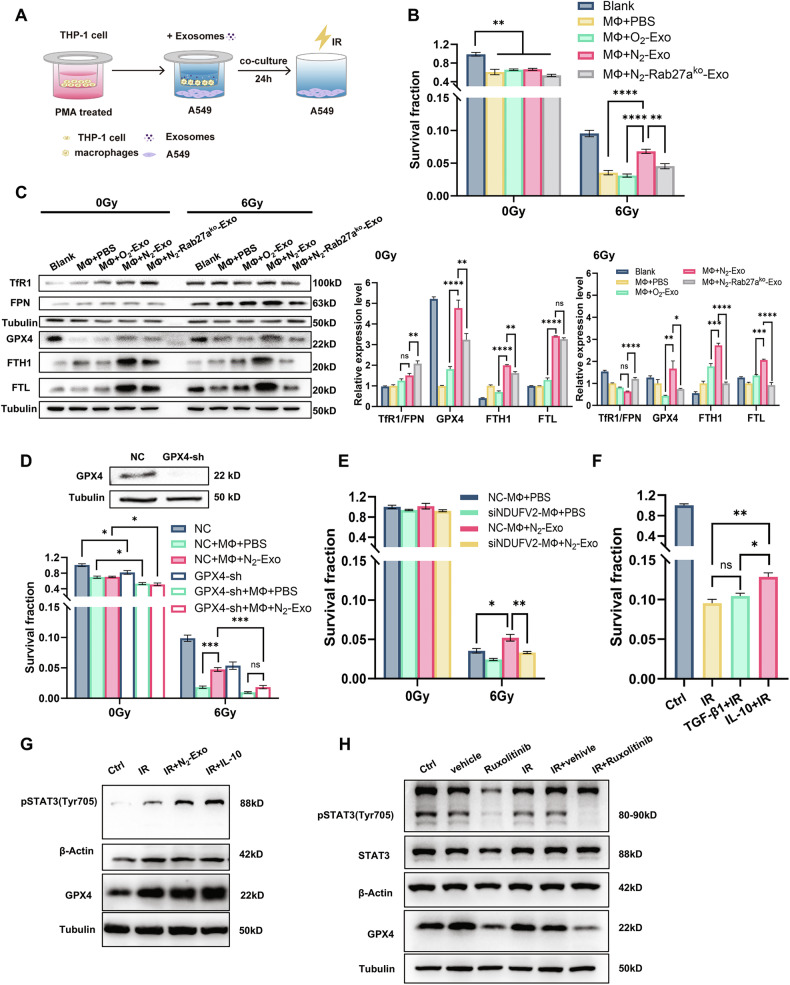
Hypoxic exosomes and macrophages mediated radioresistance of bystander normoxic A549 cells through ferroptosis pathway. **A** Schematic diagram of co-culturing THP-1 cells, exosomes, and A549 cells. THP-1 cells were pretreated with 100 ng/ml PMA for 24 h and then co-cultured with PBS, O_2_-exo, N_2_-exo or N_2_-Rab27a^ko^-exo (upper chamber) and normoxic A549 cells (lower chamber) for 24 h, then A549 cells were exposed to 6 Gy of X-ray. **B** Clonogenic survivals of A549 cells under the above treatments. Blank, without co-culture. **C** Western blot analysis of TfR1, FPN, GPX4, FTH1, and FTL in A549 cells at 6 h after IR with the above pretreatments. **D** Clonogenic survivals of 6 Gy irradiated A549 cells transfected with GPX4-sh or its negative control (NC) that were pre-cocultured with N_2_-exo and THP-1 cells for 24 h. **E** Clonogenic survivals of 6 Gy irradiated A549 cells that were pre-cocultured with N_2_-exo and THP-1 cells transfected with siNDUFV2 for 24 h. **F** Clonogenic survivals of A549 cells that were pre-treated with PBS, 1000 pg/ml TGF-β1, or 1000 pg/ml IL-10 for 24 h followed by 6 Gy irradiation. **G** Western blot of pSTAT3(Tyr705) and GPX4 expression in irradiated A549 cells treated with N_2_-exo or 1000 pg/ml IL-10. **H** Western blot of pSTAT3 and GPX4 expression in normoxic A549 cells treated with 20 nM Ruxolitinib. *P < 0.05; **P < 0.01; ***P < 0.001; ****P < 0.0001; ns no significance.

Our previous study has demonstrated that hypoxic exosomes could mediate radioresistance of bystander normoxic NSCLC cells [27]. Given the high abundance of macrophages in TME, it is practically interesting to know whether exosome-induced macrophage polarization modulates the radiosensitivity of bystander NSCLC cells. To address this, we established a transwell co-culture system containing A549 cells in the lower chamber, and exosomes together with THP-1 cells in the upper chamber (Fig. 4A). After 24 h of co-culture, A549 cells were irradiated for survival assay. Although macrophages alone reduced the colony formation rate of both nonirradiated and irradiated A549 cells, the survival of irradiated A549 cells were significantly reversed after co-culturing with N_2_-exo and macrophages, which was not observed in the groups of O_2_-exo and N_2_-Rab27a^ko^-exo (Fig. 4B). Dose–response curves also confirmed that A549 cells exhibited enhanced radioresistance after co-culture with macrophages and N_2_-exo (Fig. S5A), whereas this radioprotective effect of N_2_-exo was absent in H1299 cells (*p*53 null) (Fig. S5B, C), consistent with our previous report that the radiation-induced bystander effect was p53-dependent [34].

Based on the previous work [35], ferroptosis deficiency contributes to tumor radioresistance. We found that the expressions of the negative regulatory proteins of ferroptosis, GPX4, FTH1, and FTL, were obviously increased in A549 cells after co-culturing with macrophages treated with N_2_-exo, compared with PBS, O_2_-exo, or N_2_-Rab27a^ko^-exo treatment, although they were decreased by IR (Fig. 4C). The TfR1/FPN ratio of irradiated A549 cells pretreated with N_2_-exo + MФ had no significant difference with MФ + O_2_-exo treatment but it was much lower than that of MФ + PBS or MФ + N_2_-Rab27a^ko^-exo treatment. Moreover, we detected the changes of ferroptosis-related proteins in the subcutaneous xenograft tumor tissues, and found that the expressions of GPX4, FTH1 and FTL in the N_2_-exo group were significantly higher than those in other exosome-treated groups (Fig. S6). Depletion of macrophages in mice partially reduced the expressions of these ferroptosis suppressors in irradiated xenografts, confirming that macrophages mediated N_2_-exo-induced upregulation of ferroptosis-negative regulators in vivo.

To determine whether ferroptosis is functionally involved in hypoxia-derived exosome-mediated radioresistance, lentivirus-delivered GPX4 shRNA was introduced to effectively knockdown GPX4 in A549 cells (Fig. 4D). Importantly, co-culture with N₂-exo and macrophages failed to significantly increase the survival rate of irradiated GPX4-knockdown (GPX4-sh) A549 cells (Fig. 4D), confirming that tumor cell ferroptosis was critical for the radioprotective effect. Considering the key role of NDUFV2 in macrophage polarization and function, we next co-cultured normoxic A549 cells with NDUFV2-knockdown macrophages and found that N_2_-exo did not increase the survival of irradiated A549 cells (Fig. 4E), indicating that macrophage NDUFV2 was required for the radioprotective effect. Since N₂-exo polarized macrophages toward an M2 phenotype, we hypothesized that anti-inflammatory cytokines TGF-β1 and IL-10 might contribute to the bystander radioprotective response. To test this, we pretreated A549 cells with TGF-β1 or IL-10 prior to IR. Only IL-10, but not TGF-β1, significantly increased the survival of irradiated A549 cells (Fig. 4F), consistent with our earlier finding that N₂-exo (but not N₂-Rab27aᵏᵒ-exo) promoted IL-10 secretion from macrophages (Fig. 1G). We further explored the downstream signaling of IL-10 and found that IL-10 treatment markedly enhanced STAT3 phosphorylation (pSTAT3) and GPX4 expression in irradiated A549 cells, mimicking the effects of N₂-exo (Fig. 4G). Conversely, inhibition of IL-10/IL-10RA downstream signaling with the JAK inhibitor Ruxolitinib abolished the induction of pSTAT3 and GPX4 in normoxic A549 cells (Fig. 4H). These results demonstrated that IL-10 might confer radioresistance to bystander normoxic A549 cells through STAT3 phosphorylation and subsequent suppression of ferroptosis.

### Hypoxic exosomes promoted tumor progression and radioresistance in vivo through immunosuppressive macrophages

**Fig. 5:**
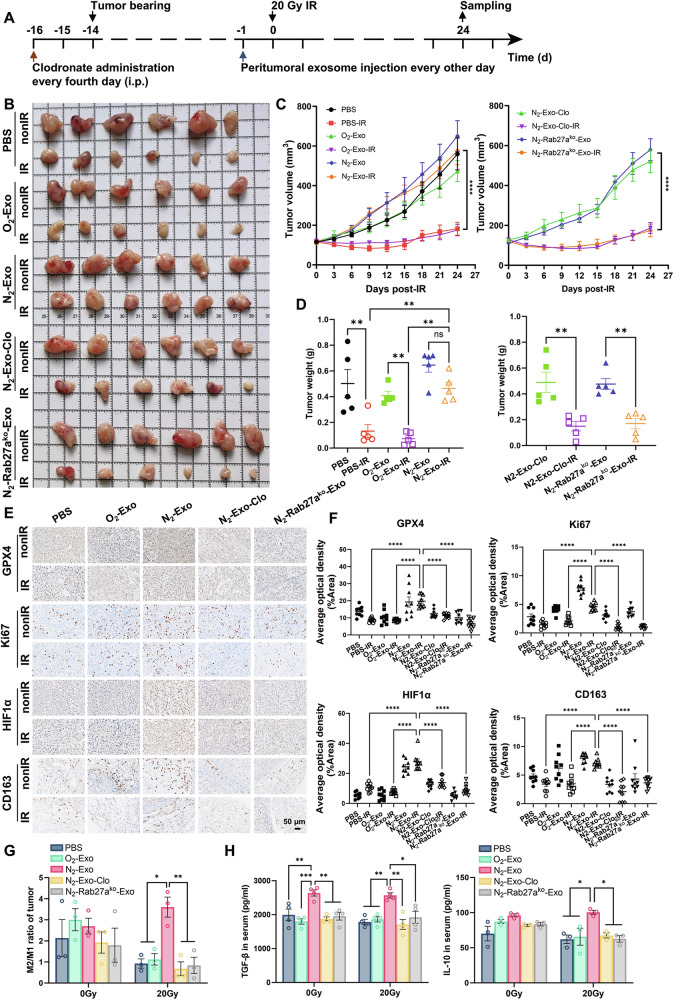
Hypoxic exosomes promoted tumor radioresistance in vivo. **A** A schematic diagram summarizing the xenograft establishment. **B** Representative xenograft tumor mass (*n* = 6) of A549 xenografts peritumorally injected with O_2_-exo, N_2_-exo, or N_2_-Rab27a^ko^-exo and then exposed to 20 Gy irradiation. **C** Volumes of A549 xenograft tumors with the indicated treatments at different days after irradiation. **D** Tumor weights of A549 xenografts of above groups on the day of sampling. **E**, **F** Representative images and quantification of immunohistochemical staining (GPX4, Ki67, HIF1α, and CD163) of A549 xenograft tumor sections with the indicated treatments. Scale bar, 50 µm. **G** Flow cytometry analysis of CD86 and CD163 showing the ratio of M2/M1 in A549 xenograft tumors (*n* = 3) with the indicated treatments. **H** ELISA analyses of TGF-β1 and IL-10 in the mice serum collected from the indicated groups. *P < 0.05; **P < 0.01; ***P < 0.001; ****P < 0.0001; ns no significance.

To further investigate how macrophages affect tumor radiosensitivity in vivo, we established a subcutaneous A549 xenograft model using male nude mice (Fig. 5A). Macrophages in mice were depleted with clodronate 2 days before tumor cell implantation, achieving >90% clearance efficiency in mice spleens and >70% in tumors (Fig. S7A). Multiplex immunohistochemistry (mIHC) of tumor sections confirmed that dendritic cells (DCs) and B cells were sparsely distributed in tumor tissues, with no significant differences between macrophage-depleted and control groups (Fig. S7B), indicating minimal involvement of other immune cell populations in the observed effects. IR significantly inhibited tumor growth in mice treated with O_2_-exo, N_2_-exo + Clo, or N_2_-Rab27a^ko^-exo, but it failed to inhibit the growth of tumors treated with N_2_-exo (Fig. 5B, D). Meanwhile, immunohistochemical assay revealed that N_2_-exo treatment significantly increased the expression levels of GPX4, Ki67, HIF1α, and CD163 in the irradiated tumors (Fig. 5E, F), and this upregulation was significantly abrogated by macrophage depletion or inhibition of exosome secretion under hypoxic conditions in vivo.

We further validated the hypoxic nature of TME by examining carbonic anhydrase IX, a well-characterized hypoxia marker, which was markedly upregulated in N_2_-exo-treated tumors (Fig. S8A). Analysis of the GEPIA2021 dataset [36] showed that high HIF1α expression predicted poor prognosis in LUAD patients (Fig. S8B). Using the TIMER2.0 online tool [37], we found a significant positive correlation between HIF1α and Rab27a expression in NSCLC tissues (Fig. S8C), suggesting N_2_-Rab27a^ko^-exo might be less effective than N_2_-exo in upregulating HIF1α in tumors. Likewise, the expression of HIF1α positively correlated with the number of infiltrating M2 macrophages (Fig. S8D), reinforcing that M2-polarized macrophages predominated in the hypoxic TME and contributed to the radioresistance. Consistently, N_2_-exo treatment significantly increased HIF1α expression in macrophages compared to O_2_-exo or N_2_-Rab27a^ko^-exo (Fig. S8E).

To further determine whether N_2_-exo orchestrated an immunosuppressive tumor microenvironment, we analyzed the intratumoral distribution of M1 and M2 macrophages, as well as serum levels of the anti-inflammatory cytokines of TGF-β1 and IL-10. Compared with the treatment with other exosomes, N_2_-exo treatment disrupted the radiation-induced immunostimulatory response, as evidenced by a significant increase in the intratumoral M2/M1 macrophage ratio following IR (Fig. 5G). Meanwhile, in the irradiated mice, N_2_-exo treatment also elevated serum TGF-β1 and IL-10, but this tendency was reversed by either macrophage depletion or Rab27a knockout (Fig. 5H). Collectively, these findings confirmed that macrophages played a critical role in N_2_-exo-mediated promotion of tumor radioresistance by fostering an immunosuppressive TME under in vivo hypoxic conditions.

### The role of ferroptosis in hypoxic exosome-mediated radioresistance of normoxic NSCLC

**Fig. 6:**
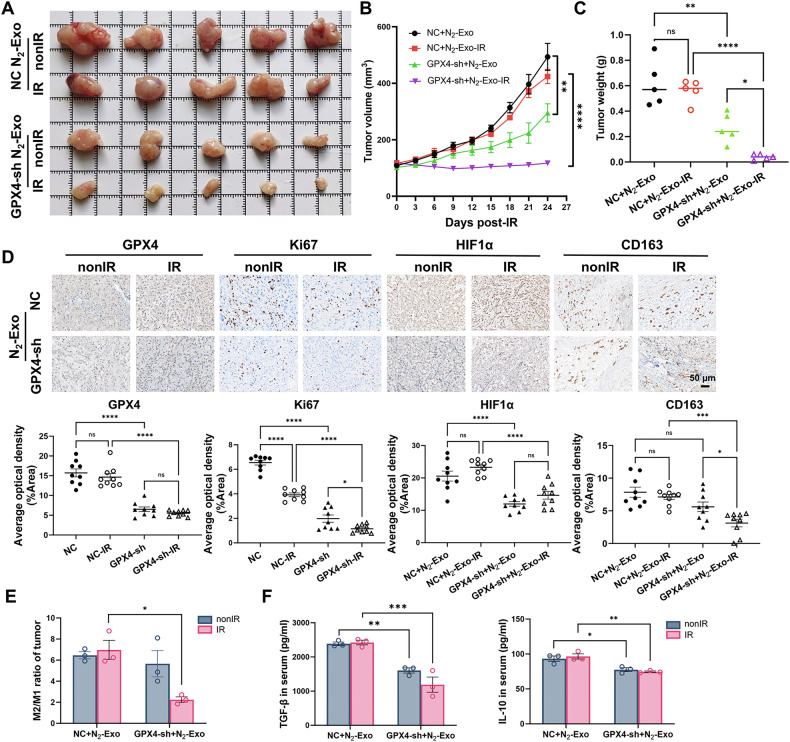
The role of ferroptosis in hypoxic exosome-mediated radioresistance of normoxic tumor cells. **A**, **B** Representative xenograft tumor mass (*n* = 5) and the tumor growth curves of A549 cells transfected with GPX4-sh or GPX4-sh-NC exposed to local irradiation of 20 Gy. All the tumors were peritumorally injected with N_2_-exo every other day starting from the day before irradiation until the mice were sacrificed. **C** Tumor weights of A549 xenografts of above groups at the day of sampling. **D** Representative images and quantification of immunohistochemical staining (GPX4, Ki67, HIF1α, and CD163) of xenograft tumors of the above groups. Scale bar, 50 µm. **E** Flow cytometry analysis of CD86 and CD163 showing the ratio of M2/M1 in the above xenograft tumors (*n* = 3). **F** ELISA analyses of TGF-β1 and IL-10 in the mice serum collected from the indicated groups. *P < 0.05; **P < 0.01; ***P < 0.001; ****P < 0.0001; ns no significance.

So far, our findings have demonstrated that hypoxic exosomes upregulated the expression of GPX4 in subcutaneous tumors via macrophages, thereby mediating radioresistance in A549 xenografts through ferroptosis inhibition. To further clarify the role of ferroptosis in this macrophage-regulated, exosome-mediated radioresistance, we established subcutaneous A549 xenografts with stable GPX4 knockdown. It was found that GPX4 silencing reversed the radioresistance induced by peritumoral injection of N_2_-exo (Fig. 6A–C). Immunohistochemical analysis revealed that GPX4 knockdown reduced the expressions of Ki67, GPX4, HIF1α, and CD163 in tumors regardless of IR (Fig. 6D), suggesting that GPX4 silencing hindered tumor cell proliferation, alleviated tumor hypoxia, and decreased M2-polarized macrophage infiltration. Consistent with this, the reduced M2/M1 ratio of infiltrating macrophages in the irradiated tumors further supported the hypothesis that lipid peroxidation accumulation, a hallmark of ferroptosis, might shift infiltrating macrophages from M2 to M1 (Fig. 6E). In addition, the secretion of immunosuppressive factors TGF-β1 and IL-10 in mice serum were significantly reduced by GPX4 knockdown in xenografts (Fig. 6F). Taken together, these results highlighted that GPX4 inhibition enhanced radiosensitivity and modulated immunoreactive responses in N_2_-exo-treated xenografts.

**Fig. 7:**
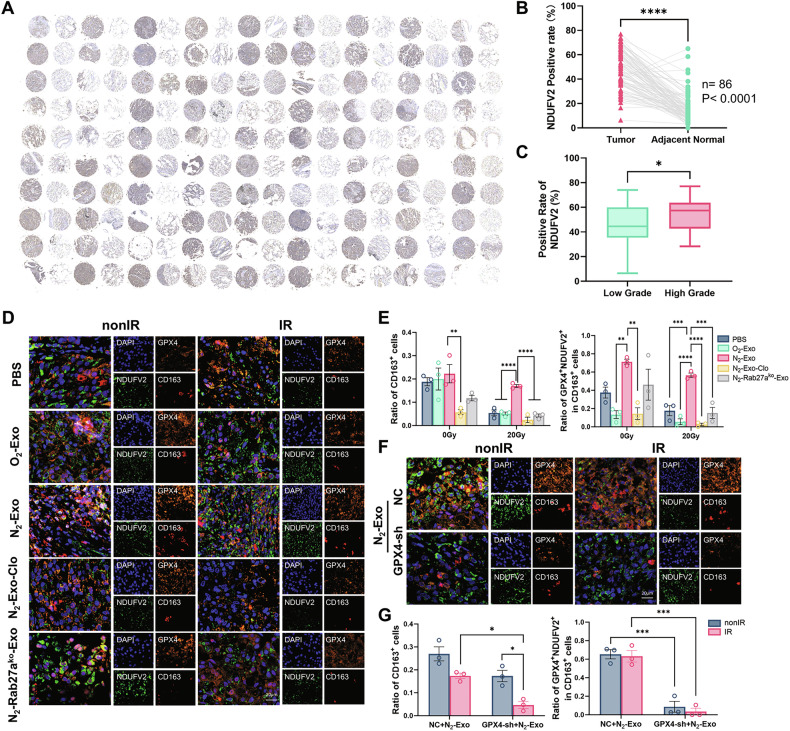
Clinical relevance of NDUFV2 in LUAD and its colocalization with GPX4^+^CD163^+^ macrophages in xenografts. **A** Tissue microarray of clinical LUAD samples (Cat no. HLugA180Su12) showing NDUFV2 in tumor tissues and paired adjacent normal tissues. **B** Positive rate of NDUFV2 expression in tumor versus adjacent normal tissues, *n* = 86. **C** Comparison of NDUFV2 positivity between low-grade (*n* = 61) and high-grade (*n* = 25) tumors. **D**, **E** Representative images of multiplex immunofluorescence staining in paraffin-embedded xenograft tumors for GPX4 (orange), NDUFV2 (green), CD163 (red), and DAPI (blue). Scale bar, 20 µm. **F**, **G** The ratio of CD163^+^ cells and GPX4^+^NDUFV2^+^CD163^+^ cells in the representative fields (n = 3) of tumor sections of the indicated groups. *P < 0.05; **P < 0.01; ***P < 0.001; ****P < 0.0001; ns no significance.

Moreover, we validated the clinical relevance of NDUFV2 using tissue microarray analysis (HLugA180Su12). Results showed that NDUFV2 expression was significantly higher in NSCLC tumor tissues than in paired adjacent normal tissues, and NDUFV2 expression was positively correlated with advanced pathological stages (Fig. 7A–C). Because NDUFV2 enhanced M2 polarization and ferroptosis inhibition of macrophages after N_2_-exo treatment, we wondered whether NDUFV2 and GPX4 exhibited correlative expression pattern in intratumoral infiltrating M2-polarized macrophages in vivo. Multiplex fluorescent immunohistochemical staining (mIHC) revealed that N_2_-exo treatment markedly enhanced the expression of GPX4, NDUFV2, and CD163 (M2 marker) of intratumoral macrophages (Fig. 7D, E). Moreover, in the subcutaneous tumors with GPX4 knockdown, the expressions of GPX4, NDUFV2, and CD163 failed to be increased even after N_2_-exo treatment (Fig. 7F, G). Collectively, these results indicated that the hypoxic exosome treatment could preferentially recruit M2-polarized macrophages with high expression levels of NDUFV2 and GPX4 into the tumor microenvironment.

## Discussion

The interplay between NSCLC cells and immune cells within TME exerts a critical influence on cancer progression and responsiveness to radiotherapy. Localized intratumoral hypoxia and cellular ferroptosis suppression are key factors contributing to the poor efficacy of NSCLC radiotherapy, during which macrophages play a critical role. Our study demonstrated that hypoxic NSCLC cell-derived exosomes drove radioresistance in bystander normoxic NSCLC cells through ferroptosis inhibition, regulated by macrophages. Mechanistically, N_2_-exo induced M2 polarization of macrophages and promoted their mitochondrial functions via upregulation of NDUFV2, ultimately leading to suppressed ferroptosis in macrophages. But macrophages with NDUFV2-knockdown lost the ability to transmit hypoxia-induced radioresistance signals to bystander normoxic tumor cells.

Exosomes are well-recognized as key messengers in hypoxic TME [38]. In our previous work, we analyzed the proteomic differences between exosomes derived from normoxic and hypoxic NSCLC cells and identified 130 upregulated and 129 downregulated proteins in N_2_-exo. Further Gene Ontology (GO) enrichment analysis revealed distinct profiles of these differentially expressed proteins (DEPs) across three functional categories: biological process, cellular component, and molecular function (Table S1). Among those DEPs, angiopoietin-like protein 4 (ANGPTL4) protein was found to be enriched in hypoxic exosomes and contributed to radioresistance of NSCLC cells [27]. Intriguingly, our other study noted that A549 cells treated with exosomes from ANGPTL4-knockdown hypoxic NSCLC cells exhibited higher migration ability than those treated with normoxic exosomes [39], suggesting functional complementarity between different exosomal cargoes. Solid tumors exhibit significant heterogeneity in exosome quantity, content, size, and function under normoxic versus hypoxic conditions [40]. In the present study, we standardized exosome quantities across all experimental groups to eliminate dose-dependent biases. Our results showed that while hypoxic NSCLC cells secreted a higher number of exosomes per cell than normoxic cells, normoxic exosomes were larger in size but had similar total protein content to hypoxic exosomes. These observations indicated that the divergent effects of N_2_-exo and O_2_-exo on macrophage function likely stemmed, in part, from differences in their cargo of signaling molecules. Notably, we confirmed that NDUFV2 was enriched in hypoxic exosomes and that its expression was elevated in recipient macrophages following exosome uptake. The mechanism underlying hypoxia-induced NDUFV2 enrichment in exosomes remains incompletely understood, and it may involve alterations in exosome biogenesis, cargo sorting mechanisms, or other upstream regulatory processes that require further investigation.

Plenty of evidence indicates that exosomes secreted from hypoxic tumors carry specific proteins, lipids, and RNAs (miRNAs, lncRNAs, and circRNAs), which mediate TAM recruitment and M2 polarization [10]. Herein, we investigated the potential mechanism by which hypoxic tumor exosomes coordinated macrophage phenotypes, and found that incubation with N_2_-exo, but not O_2_-exo, polarized macrophages toward an M2 phenotype. This was evidenced by a higher M2/M1 macrophage ratio in the cell population and increased secretion of the anti-inflammatory factors TGF-β1 and IL-10 into the culture medium (Fig. 1). An elevation in OCR suggested that N_2_-exo influenced the mitochondrial function of macrophages via NDUFV2, characterized by increased intracellular levels of ATP and MMPs (Fig. 2). Additionally, N_2_-exo significantly enhanced macrophage phagocytosis, consistent with the finding that M2-type macrophages exhibited a higher phagocytic capacity than M1-type macrophages [41]. Furthermore, N_2_-exo upregulated HIF-1α protein expression in macrophages (Fig. S8E), aligning with previous reports that HIF-1α enhanced macrophage phagocytic function [42]. This suggested a link between the hypoxic TME, exosome-mediated HIF-1α regulation, and macrophage phagocytosis.

NDUFV2 is a core subunit of mitochondrial complex Ⅰ, which catalyzes the oxidation of NADH to NAD^+^ and transfers electrons to coenzyme Q_10_ [43]. In macrophages with NDUFV2 knockdown, we observed a reduced M2/M1 ratio and decreased secretion of the anti-inflammatory factors TGF-β1 and IL-10. Meanwhile, iron homeostasis is known to be tightly linked to macrophage polarization [33], and our results demonstrated that N_2_-exo could suppress ferroptosis in macrophages via an NDUFV2-dependent manner (Fig. 3). A previous study reported that iron overload downregulated glia maturation factor-γ (GMFG), and GMFG inhibition reduced NDUFV2 expression [44]. This led us to hypothesize that iron ions may inhibit M2-like macrophage polarization through the GMFG-oxidative phosphorylation (OXPHOS) pathway, an area requiring further investigation. We confirmed that NDUFV2 was enriched in N_2_-exo and upregulated in recipient macrophages following exosome uptake. Compared with O_2_-exo, N_2_-exo enhanced macrophage mitochondrial function and suppressed ferroptosis; these effects were partly recapitulated by recombinant NDUFV2 protein and abolished by NDUFV2 knockdown. These findings supported a dual mechanism for NDUFV2 elevation in macrophages: it arose from both exosomal transfer and transcriptional activation of NDUFV2 in recipient cells. The upstream signals responsible for transcriptional regulation of NDUFV2 were not investigated here, but hypoxia-inducible factors [45], mitochondrial regulators such as Nrf1 [46], and possible epigenetic mechanisms (e.g., histone modifications) [47] are plausible candidates for future investigation.

Beyond NDUFV2, other electron transport chain (ETC) components have also been implicated in ferroptosis and anti-tumor therapy response. For instance, complex I subunit NDUFS4 [48], complex II (which regulates lipid ROS) [49], and complex V (which maintains bioenergetics and ferroptosis sensitivity) [50] have all been linked to these processes. While our findings highlighted NDUFV2 as a pivotal mediator of macrophage-driven ferroptosis inhibition, future studies comparing the roles of other ETC subunits will help clarify whether NDUFV2 acts uniquely or as part of a broader mitochondrial regulatory network.

Radioresistance mediated by cargoes of hypoxic tumor exosomes has been extensively studied [51]. However, the role of macrophages, innate immune cells with the highest infiltration rate in TME [52], in hypoxia-induced radioresistance remains poorly understood. The present study demonstrated that N_2_-exo induced M2 polarization and ferroptosis inhibition in macrophages could benefit the survival of pro-tumorigenic TAMs, thus promoting tumor growth and radioresistance. In a tripartite co-culture system containing exosomes, macrophages, and normoxic A549 cells, N_2_-exo significantly inhibited ferroptosis in the bystander normoxic cells, ultimately enhancing their radioresistance (Fig. 4B, C). Mechanistically, N_2_-exo reduced intracellular ferrous ion (Fe^2+^) levels in these bystander cells by increasing iron storage, an effect mediated by decreasing the TfR/FPN ratio. This reduction in free Fe^2+^ minimized Fenton reaction-driven ROS generation, thereby protecting bystander tumor cells from ferroptosis.

On the other hand, N_2_-exo-treated macrophages failed to protect bystander H1299 cells (*p*53 null) from radiation damage (Fig. S5B, C), consistent with our previous report that radiation-induced bystander effect mediated by macrophages was *p*53-dependent [34]. Inflammatory cytokines associated with RIBE are thought to be secreted by macrophages recruited to radiation-induced dead or damaged cells, recent evidence indicates that radiation triggers sustained activation of the p53 pathway, which in turn modulates macrophage functions [53, 54]. This further supported a mechanistic link between p53 signaling and macrophage-mediated bystander effects, prompting us to use A549 cells (wt*p*53)-based xenografts for further in vivo experiments. Our results identified TGF-β1 and IL-10, particularly IL-10, as key mediator in the crosstalk among hypoxic exosomes, macrophages, and bystander normoxic tumor cells (Fig. 4F). IL-10 engages the JAK1/STAT3 signaling pathway, in which receptor-associated JAK1 phosphorylates STAT3 to regulate its downstream targets such as GPX4 [55]. Consistent with this, we found that IL-10 treatment enhanced pSTAT3 and GPX4 expression in irradiated A549 cells, whereas inhibition of JAK1 with Ruxolitinib attenuated these responses (Fig. 4G, H). Our findings implied that the IL-10/STAT3 signaling axis served as a mechanistic link between macrophage-derived IL-10 to ferroptosis suppression and subsequent radioresistance in bystander normoxic tumor cells.

Our in vivo studies demonstrated that peritumoral injection of N_2_-exo reduced the radiosensitivity of A549 xenografts, which was accompanied by increased expressions of GPX4, Ki67, HIF1α, and CD163 (Fig. 5B–F). By mitigating lipid peroxidation and inhibiting ferroptosis, GPX4 forms part of a negative feedback loop to blunt tumor radiosensitivity [20]. The increase of HIF1α expression in irradiated xenografts likely reflects localized intratumoral hypoxia induced by radiation, as radiation can disrupt tumor vasculature and exacerbate oxygen deprivation. Notably, peritumoral injection of N_2_-Rab27a^ko^-exo failed to induce xenograft radioresistance. This lack of effect may be attributed to reduced exosome secretion (due to Rab27a deficiency) and/or altered exosomal cargo composition—consistent with previous reports that Rab27a blockade suppresses colorectal cancer progression [56], supporting Rab27a’s role as a potential oncoprotein in mediating tumor development. Therefore, Rab27a could serve as a potential therapeutic target to disrupt hypoxia-derived exosome-mediated radioresistance. Beyond targeting Rab27a, alternative therapeutic strategies can be envisioned. Leveraging the natural homing capacity of exosomes, one promising approach involves engineering exosomes loaded with NDUFV2-neutralizing antibodies to specifically target macrophages or tumor cells, thereby blocking NDUFV2’s role in ferroptosis inhibition and M2 polarization. Similarly, embedding the exosome biogenesis inhibitor GW4869 within exosomal membranes could enable localized suppression of exosome release in TME, further limiting the spread of radioprotective signals from hypoxic tumor cells to bystander cells.

Macrophages are the most abundant innate immune cells in the tumor immune microenvironment (TIME) [52]. Tissue-resident macrophages in the lung promote NSCLC invasion by expanding regulatory T cell (Treg) populations and reducing CD8^+^ T cell accumulation [57]. Our study provides the first evidence that depletion of mouse macrophages significantly abrogates N_2_-exo-mediated radioresistance, highlighting macrophages as indispensable mediators of hypoxic tumor radioresistance. Moreover, clodronate treatment did not alter the abundance of DCs or B cells in tumors (Fig. S7B), further confirming that the observed tumor radioresistance was specifically dependent on macrophages, rather than other innate immune cell populations. Interestingly, GPX4-knockdown A549 xenografts exhibited reduced recruitment of immunosuppressive macrophages (Fig. 6D, E), a finding consistent with previous reports that GPX4 inhibition not only induces tumor ferroptosis but also enhances anti-tumor immunity [58]. In line with this, tumors treated with N_2_-exo showed increased recruitment of CD163^+^ M2 macrophages, among which the proportion of cells co-expressing GPX4 and NDUFV2 (GPX4^+^NDUFV2^+^) was significantly higher (Fig. 7D–G), a signature associated with lower radiosensitivity. A prior study reported that the activation of M1 macrophage prevented M2 repolarization by inducing mitochondrial dysfunction [59]. In contrast, our findings showed that NDUFV2 inhibition in macrophages dampened mitochondrial function and promoted their repolarization toward an inflammatory M1 phenotype. Therefore, these results suggest that targeting NDUFV2 to reprogram macrophage metabolism could represent a promising therapeutic strategy to improve radiotherapy efficacy.

## Conclusions

**Fig. 8:**
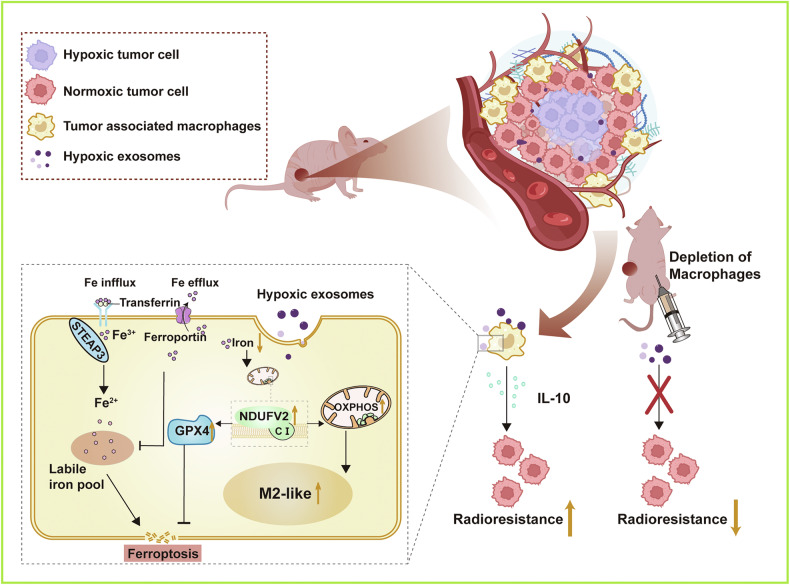
Schematic diagram for the effects of hypoxic exosomes on macrophages and radioresistance of bystander tumor cells. Hypoxic tumor cell-derived exosomes suppressed inflammatory responses and ferroptosis of macrophages via NDUFV2, which enhanced the radioresistance of bystander lung cancer cells.

This study revealed that the exosomes released from hypoxic NSCLC rendered radioresistance to bystander normoxic tumor cells by inhibiting ferroptosis mediated by macrophages that could be abundantly accumulated in hypoxic TME and had a profound impact on tumor progression and radiotherapy efficacy. Mechanistically, compared with normoxic exosomes, hypoxic exosomes significantly upregulated NDUFV2 protein expression in macrophages, thereby enhancing mitochondrial oxidative phosphorylation, which in turn suppressed the inflammatory response of macrophages. Concurrently, increase of NDUFV2 promoted the expression of GPX4, reduced intracellular iron levels, and collectively inhibiting ferroptosis in macrophages. The population of M2 polarized-TAMs with high expression level of NDUFV2 might persist in the TME due to their ferroptosis-resistant state. Macrophages secreted anti-inflammatory cytokine IL-10, which acted as a key messenger in transmitting radioresistance to bystander normoxic tumor cells by inhibiting ferroptosis pathways. Critically, macrophage depletion markedly abrogated hypoxic exosome-induced tumor radioresistance (Fig. 8). In summary, we identified an essential role of TAMs in mediating tumor radioresistance under hypoxic microenvironment, where hypoxic exosomes contributed to ferroptosis inhibition and immunosuppression through NDUFV2 in macrophages. These findings highlighted promising therapeutic opportunities, targeting TAMs, exosome secretion, or tumor ferroptosis, to improve the efficacy of NSCLC radiotherapy.

## Supplementary information

Supplementary Figures and Table

Original WB Bolt Images

## Data Availability

Data presented in this study are available on request from the corresponding author upon reasonable request.
