# Supplementary material for: Hypoxic tumor exosomes suppress macrophage inflammation and ferroptosis via NDUFV2 to enhance bystander tumor radioresistance
Source: Cell Death Dis. 2025 Dec 19;17(1):109. doi: 10.1038/s41419-025-08357-7 (PMC12847814; doi:10.1038/s41419-025-08357-7)
Supplement: Supplementary file 1 — Supplementary Figures and Table [file 41419_2025_8357_MOESM1_ESM.pdf]

Hypoxic tumor exosomes suppress macrophage inflammation and ferroptosis via NDUFV2 to enhance bystander tumor radioresistance

Jialing Zhang[1,2,#], Xiaoya Jin[1,#], Maidina Abulaihaiti[1,#], Xinglong Liu[1], Liang Zeng[1], Yuqi Xiao[1], Yan Pan[1], Yang Bai[1], Yanwu Xu[3], Chunlin Shao[1,4,*], Jianghong Zhang[1,*].

1. Institute of Radiation Medicine, Shanghai Medical College, Fudan University, Shanghai 200032, China;
2. Radiotherapy Physics and Technology Center, Cancer Center, West China Hospital, Sichuan University, Chengdu 610041, China;
3. Department of Biochemistry, School of Integrative Medicine, Shanghai University of Traditional Chinese Medicine, Shanghai, 201203, China.
4. Department of Radiation Oncology, Shanghai Proton and Heavy Ion Center, Fudan University Cancer Hospital, Shanghai, 201321, China.

*** Correspondence:** clshao@shmu.edu.cn (C.S.); zjh551268@fudan.edu.cn (J.Z.)

Supplementary figures

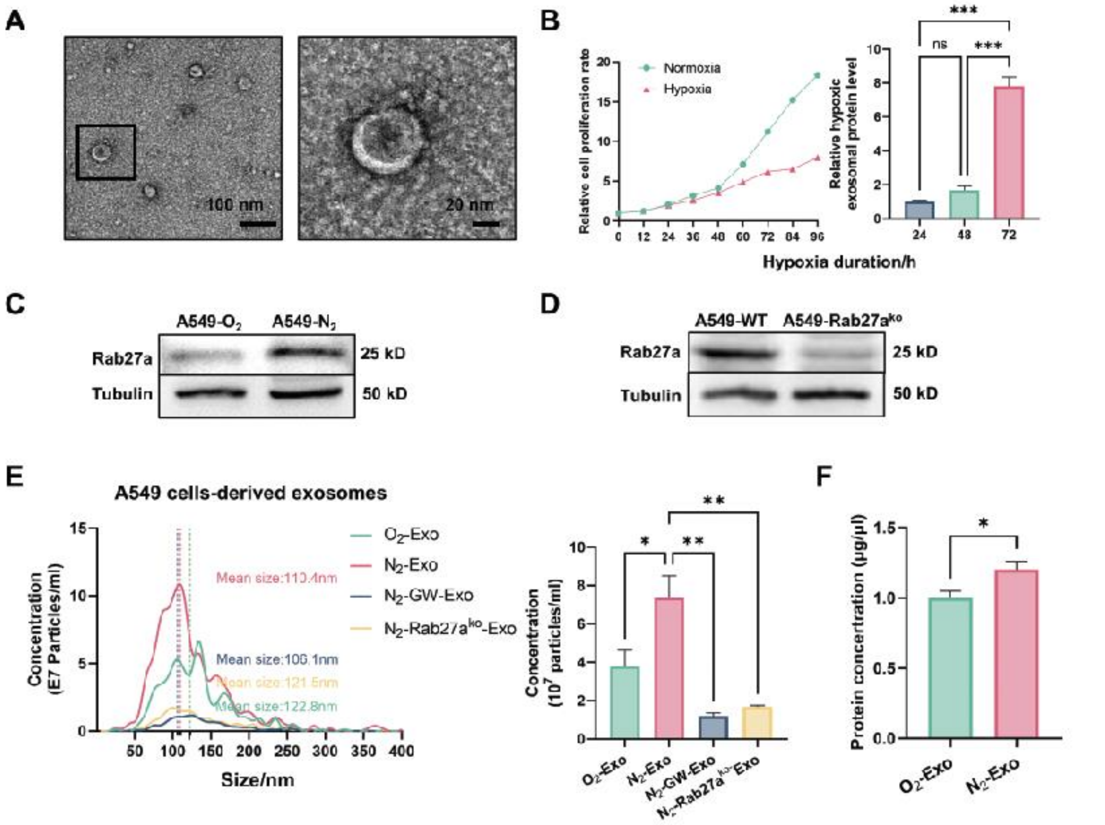

Fig. S1. Characterization of isolated exosomes and the role of Rab27a in hypoxia-induced exosome secretion. **A** Transmission electron microscopy (TEM) images of exosomes isolated from the supernatants of A549 cells. Scale bars, 100 nm (left) and 20 nm (right). **B** Relative cell proliferation rate of A549 cells under normoxic or hypoxic conditions over time (left) and quantification of exosomal protein levels in hypoxic A549 cells at different time points (right). **C** Western blot assay of Rab27a expression in A549 cells at 48 h after hypoxia treatment compared to normoxia. **D** Western blot assay of Rab27a in Rab27a-knockout A549 cells constructed by CRISPR-Cas9. **E** Particle size distributions of O_2-Exo, N_2-Exo, N_2-GW-Exo, and N_2-Rab27a^{ko}-Exo were determined by nanoparticle tracking analysis (NTA). **F** Protein concentration of O_2-Exo and N_2-Exo were determined by BCA kit. * $P < 0.05$; ** $P < 0.01$; *** $P < 0.001$.

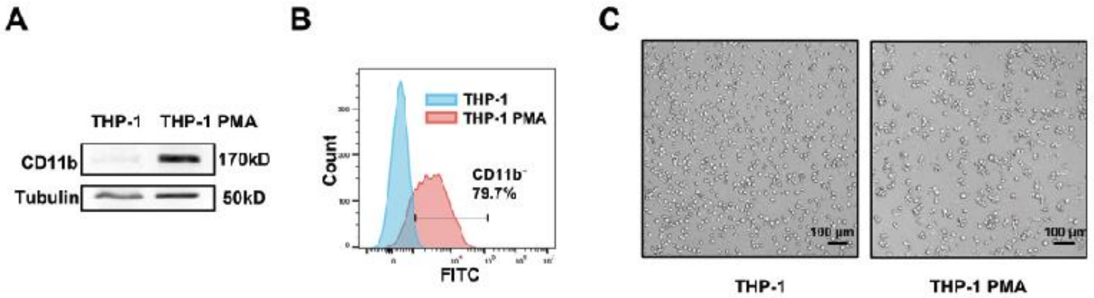

Fig. S2 PMA activated THP-1 cells into M0 macrophages. **A** Western blot assay of CD11b expression in THP-1 cells with or without 100 ng/ml PMA treatment for 24 h. **B** Flow cytometry analysis of CD11b level in THP-1 cells with or without PMA treatment. **C** Bright-field images of THP-1 cells before and after 24 h of PMA stimulation. Scale bar, 100 µm.

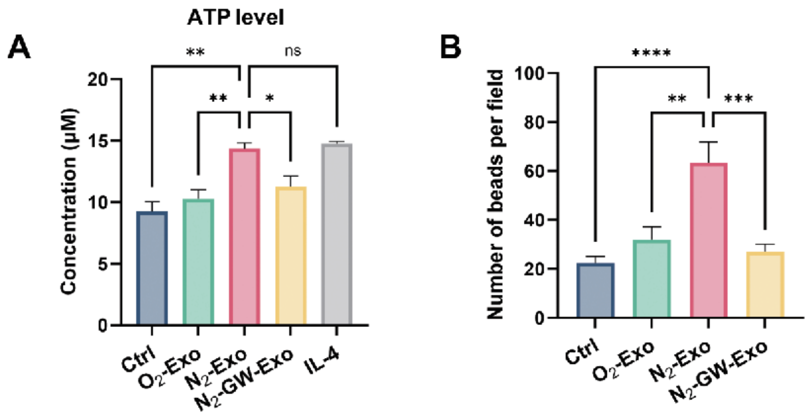

Fig. S3 The influence of H1299 cells-derived exosomes on macrophage ATP level and phagocytic capacity. A Intracellular ATP level in THP-1 cells after 24 h incubation with indicated exosomes derived from H1299 cells. 50 ng/ml IL-4 was used as a positive control of stimulating THP-1 cells into M2 macrophages, PBS as control. **B** Phagocytosis levels of THP-1 cells incubated with H1299 cells-derived exosomes for 24 h engulfing IgG-PE beads. * $P < 0.05$; ** $P < 0.01$; *** $P < 0.001$; **** $P < 0.0001$; ns, no significance.

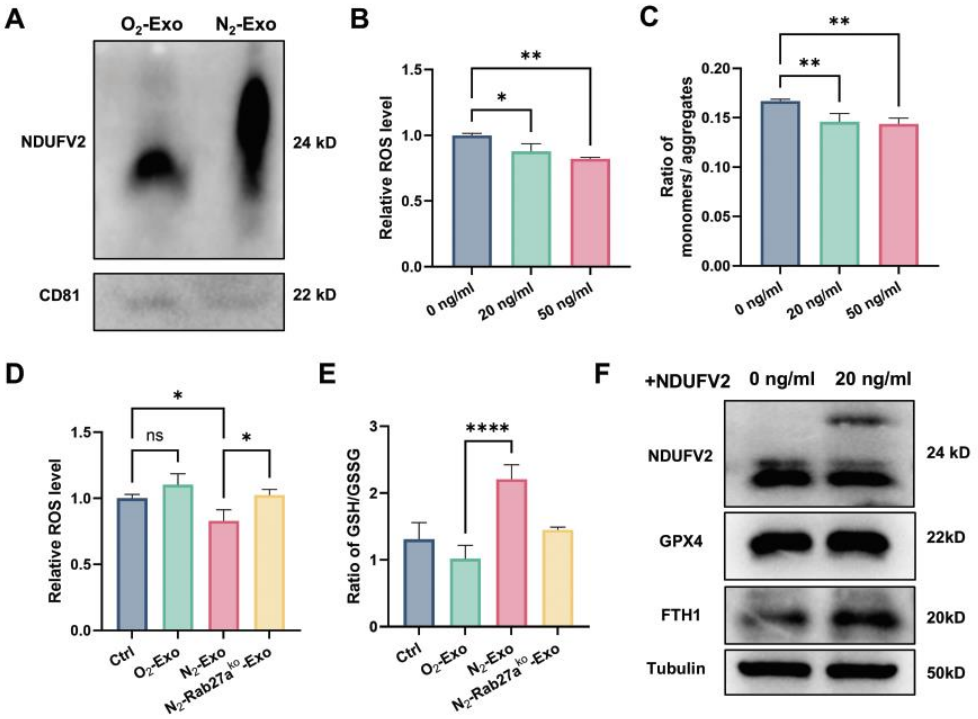

Fig. S4 Enrichment of NDUFV2 in hypoxic exosomes and its effects on mitochondrial function and ferroptosis in macrophages. A Western blot assay of NDUFV2 expression in O_2-Exo and N_2-Exo. CD81 served as an internal control. **B-C** Cellular ROS levels (B) and the ratio of JC-10 monomers/aggregates (C) in THP-1 cells after supplementation with recombinant NDUFV2 protein (0, 20, or 50 ng/ml). **D-E** Cellular ROS levels (D) and GSH/GSSG(E) ratios in THP-1 cells treated with indicated exosomes. **F** Western blot assay of NDUFV2, GPX4, and FTH1 expression in THP-1 cells with or without recombinant NDUFV2 protein.* $P < 0.05$; ** $P < 0.01$; **** $P < 0.0001$; ns, no significance.

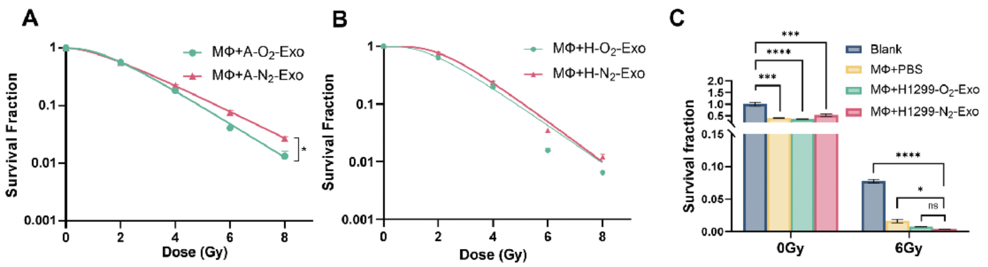

Fig. S5 The effect of exosome–macrophage co-culture on radiosensitivity of NSCLC cells. A-B Survival curves of A549 (**A**) and H1299 cells (**B**) co-cultured with indicated exosomes and THP-1 cells for 24 h followed by irradiation at 2, 4, 6, 8 Gy. **C** Clonogenic survivals of H1299 cells that were co-cultured with PBS, O_2-Exo, or N_2-Exo and THP-1 cells for 24 h followed by 6 Gy IR. Blank, without co-culture. * P< 0.05; *** P < 0.001; **** P< 0.0001; ns, no significance.

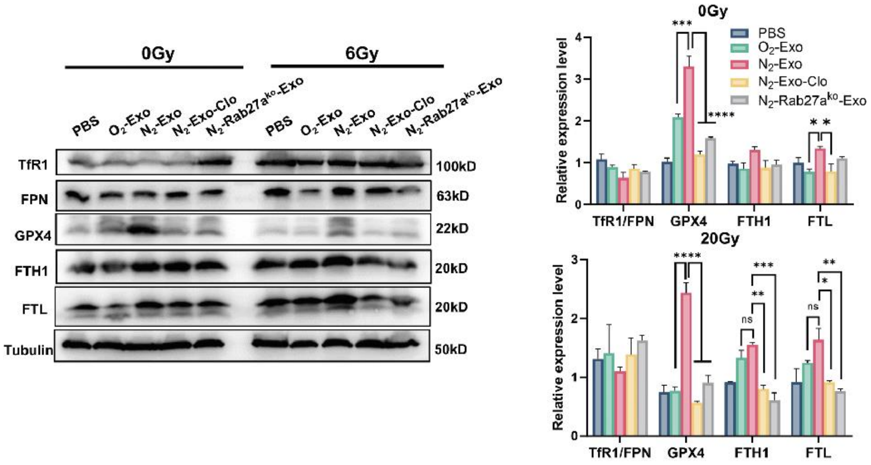

Fig. S6 Hypoxic exosomes induced ferroptosis inhibition of tumors in the presence of macrophages *in vivo*. Western blot analysis of TfR1, FPN, GPX4, FTH1, FTL, and tubulin proteins in A549 xenografts peritumorally injected with PBS, O_2-Exo, N_2-Exo, or N_2-Rab27a^{ko}-Exo with indicated treatments. * P< 0.05; ** P < 0.01; *** P < 0.001; **** P< 0.0001; ns, no significance.

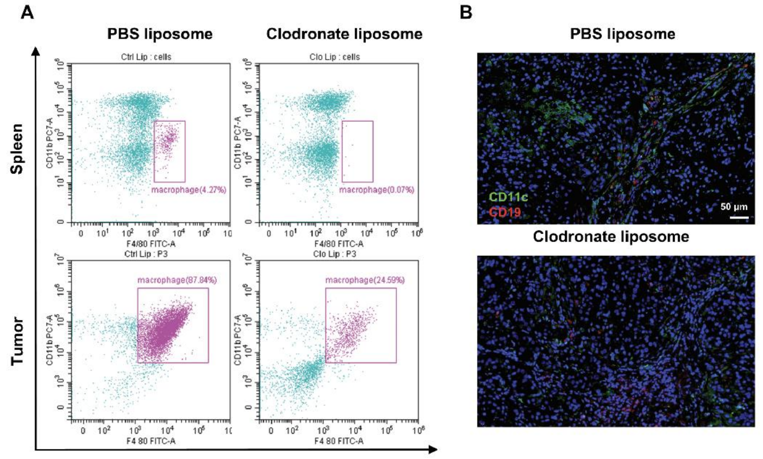

Fig. S7 Validation of macrophage depletion and evaluation of other immune cell populations in xenograft tumors. A 200 µl of clodronate liposomes (5 mg/ml) were intraperitoneally injected two days before tumor-bearing, then 100 µl of clodronate was maintained every 4 days. Flow cytometry analysis of macrophages in spleens (F4/80$^+$CD11b^{low}) and tumors (F4/80$^+$CD11b$^+$) was performed. **B** Multiplex immunohistochemistry of tumor sections showing CD11c$^+$ dendritic cells (green) and CD19$^+$ B cells (red) in control and macrophage-depleted groups. Scale bar, 50 µm.

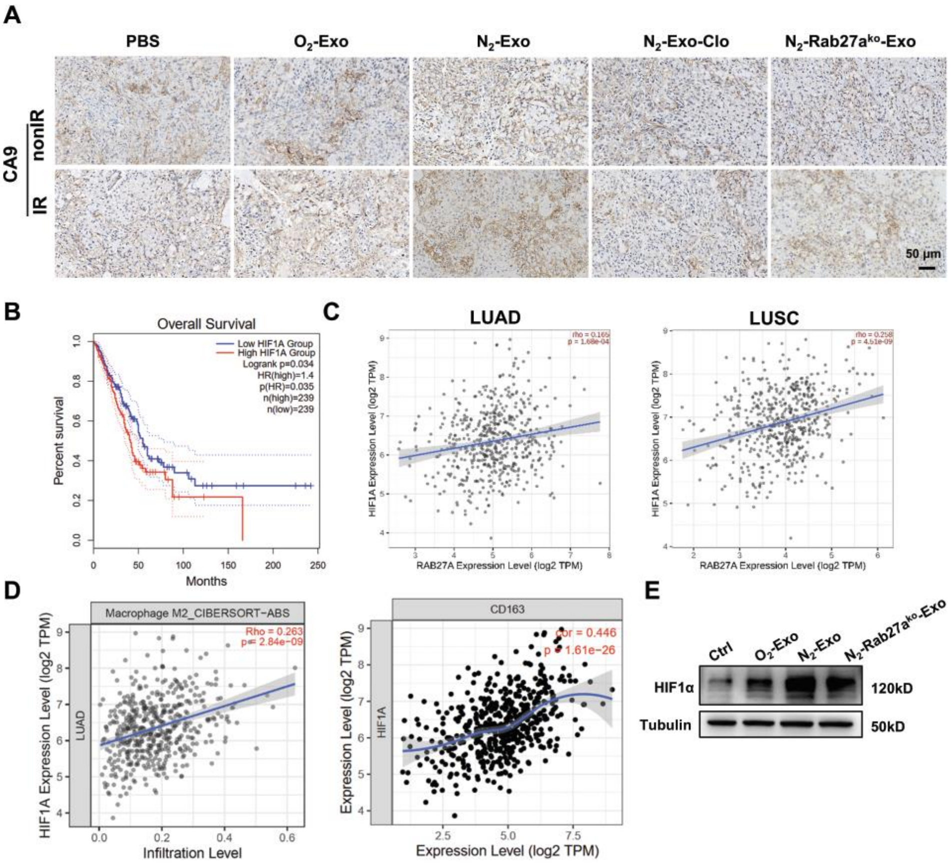

Fig. S8 Hypoxia marker validation and correlation of HIF1α with prognosis and immune features in NSCLC. A Representative images of immunohistochemical staining of CA9 in of A549 xenograft tumor sections with the indicated treatments. Scale bar, 50 μm. **B** High expression of HIF1α predicted a poor prognosis in lung adenocarcinoma based on the GEPIA2021 database. **C** Correlation analyses between the expression levels of HIF1α and Rab27a in lung adenocarcinoma (LUAD) and lung squamous cell carcinoma (LUSC) based on the TIMER2.0 online tool. **D** Correlation analysis between HIF1α expression and the number of infiltrating M2 macrophages or CD163 expression based on the TIMER2.0. **E** Western blot assay of HIF1α expression in THP-1 cells after 6 h incubation with indicated exosomes.

Table S1. GO analysis using tandem mass tag (TMT) quantitative proteomic analysis for exosome protein.

GO terms level 1	GO terms level 2	Upregulated	Downregulated
Biological Process	metabolic process	52	19
	cellular process	50	34
	single-organism process	35	27
	localization	11	13
	biological regulation	10	21
	reponse to stimulus	7	17
	signaling	5	16
	biological adhesion	3	8
	cellular component organization or biogenesis	2	10
	other	2	3
Cellular Component	cell	44	22
	macromolecular complex	21	14
	supramolecular complex	0	1
	organelle	18	16
	membrane	8	22
	extracellular region	6	9
	membrane-enclosed lumen	1	0
Molecular Function	binding	101	87
	catalytic activity	49	24
	structural molecule activity	10	10
	transporter activity	4	6
	molecular transducer activity	0	4
	molecular function regulator	2	3
	signal transducer activity	0	3
	other	0	1
