# Supplementary material for: Hypoxic tumor exosomes suppress macrophage inflammation and ferroptosis via NDUFV2 to enhance bystander tumor radioresistance
Source: Cell Death Dis. 2025 Dec 19;17(1):109. doi: 10.1038/s41419-025-08357-7 (PMC12847814; doi:10.1038/s41419-025-08357-7)
Supplement: Supplementary file 2 — Original WB Bolt Images [file 41419_2025_8357_MOESM2_ESM.pdf]

Hypoxic tumor exosomes suppress macrophage inflammation and ferroptosis via NDUFV2 to enhance bystander tumor radioresistance

Original uncropped gels of

Western blot assay

The original uncropped gels for Western blots in Figure 2.

Western blots in the article

Fig. 2E

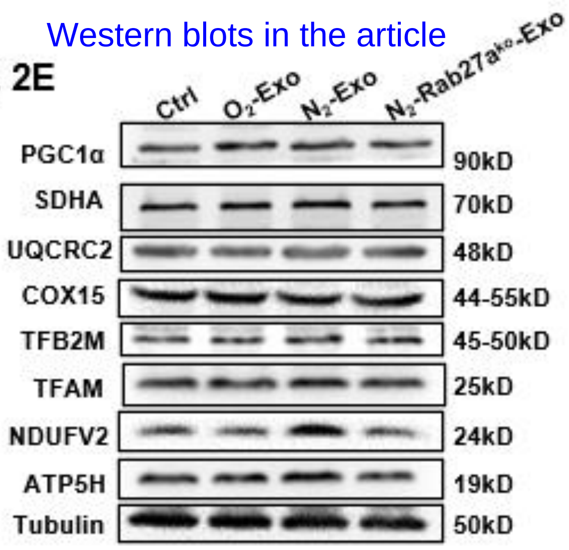

Western blots in the article

Fig. 2F

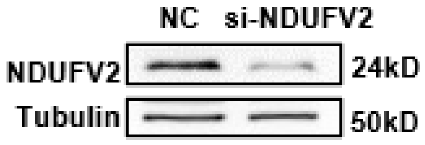

Original uncropped western blot gels

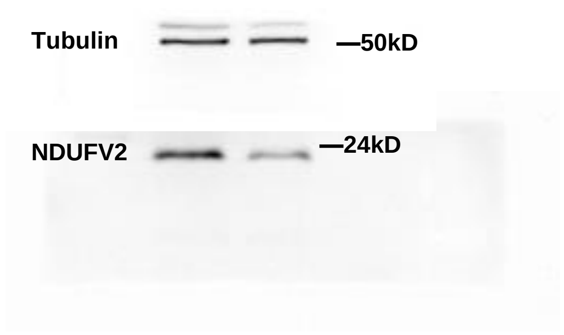

Original uncropped western blot gels

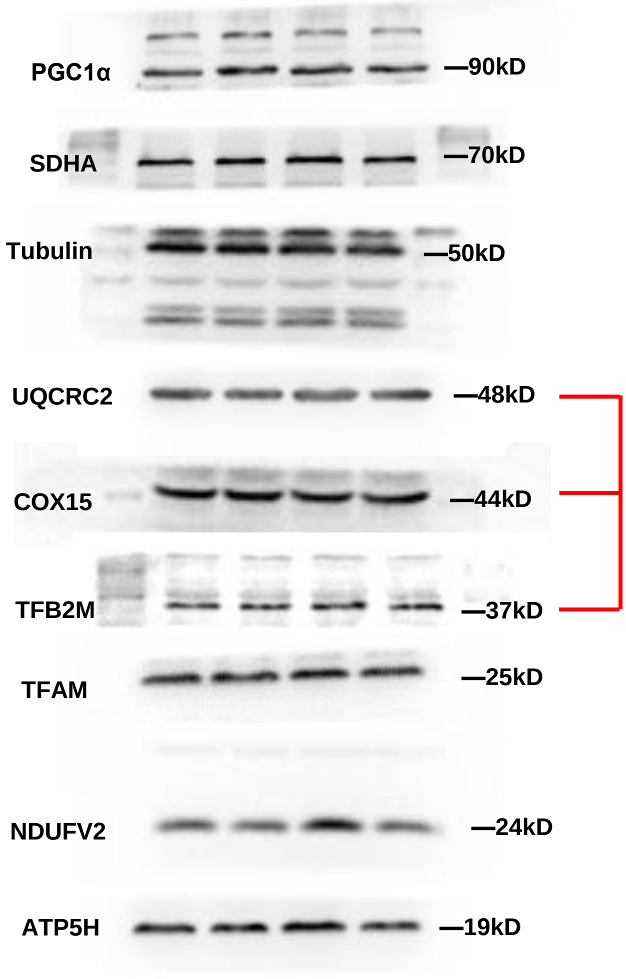

Cut from the above membrane of Tubulin
For secondary Western blot after striping

The original uncropped gels for western blots in Figure 3.

Western blots in the article

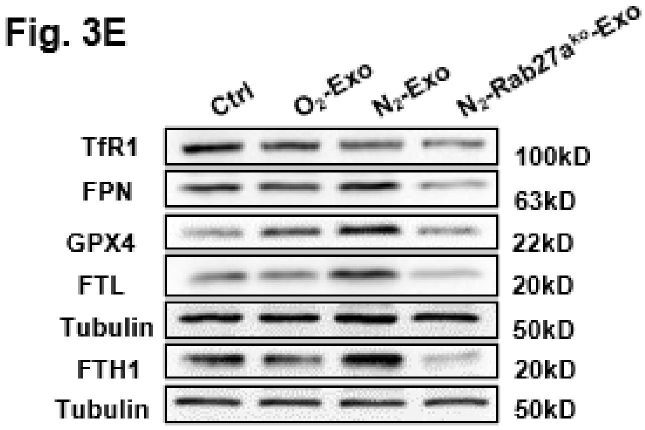

Western blots in the article

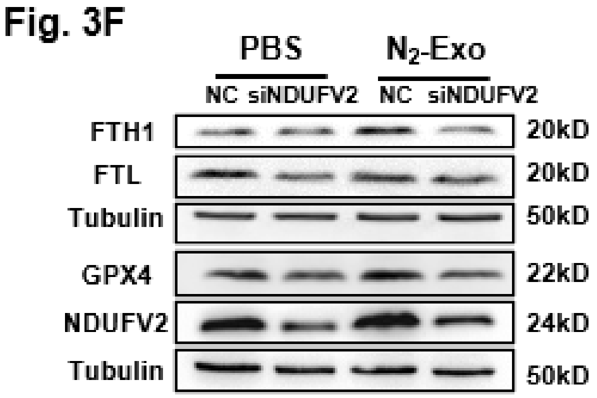

Original uncropped western blot gels

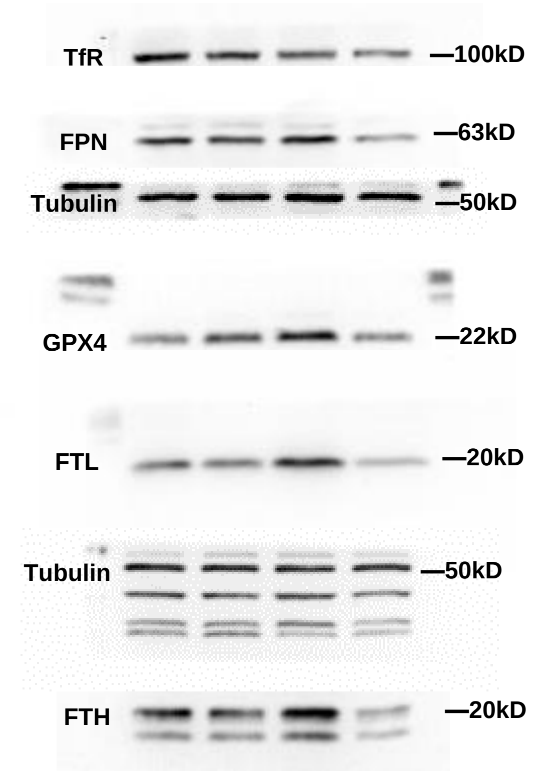

Original uncropped western blot gels

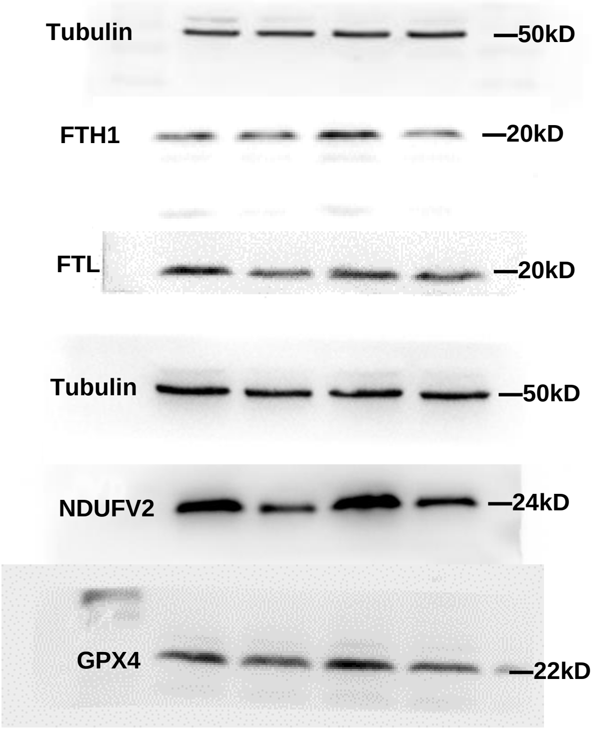

The original uncropped gels for western blots in Figure 4.

Western blots in the article

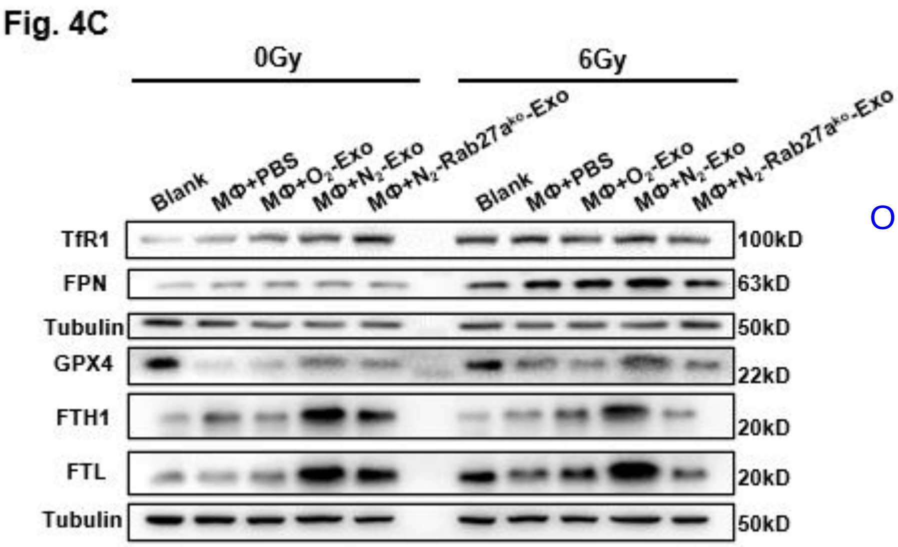

Western blots in the article

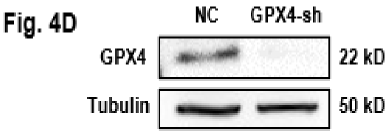

Original uncropped western blot gels

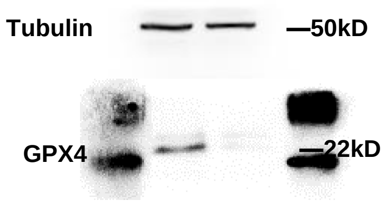

Original uncropped western blot gels

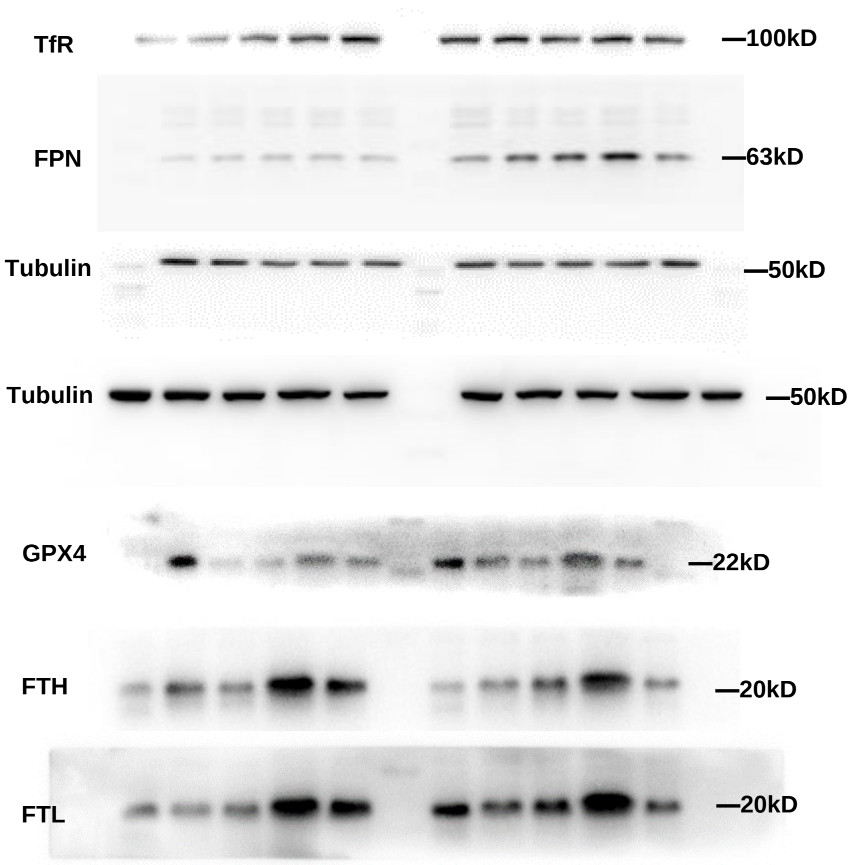

Secondary Western blot after striping with the same loading amount of proteins

The original uncropped gels for western blots in Figure 4.

Western blots in the article

Fig.4G

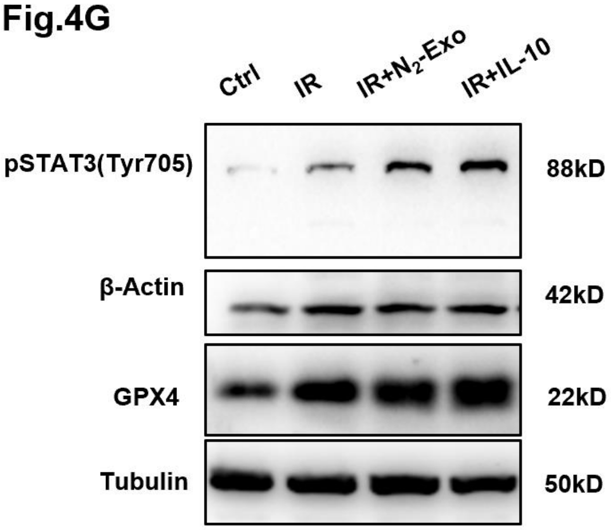

Western blots in the article

Fig.4H

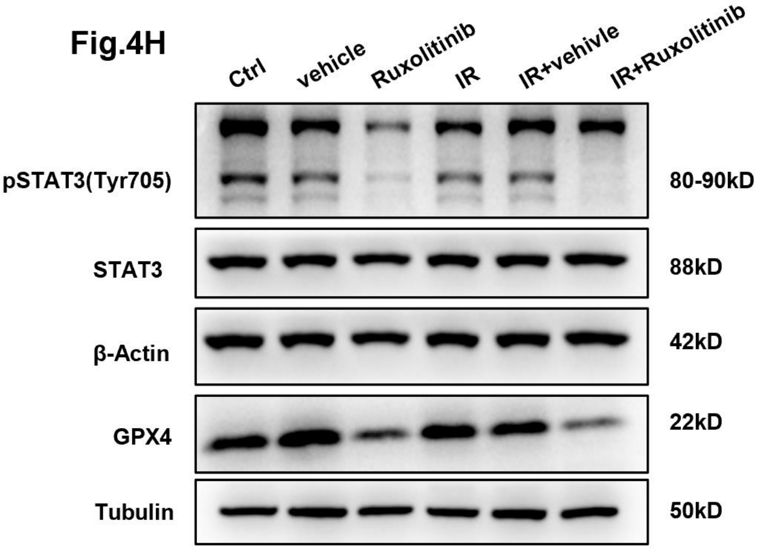

Original uncropped western blot gels

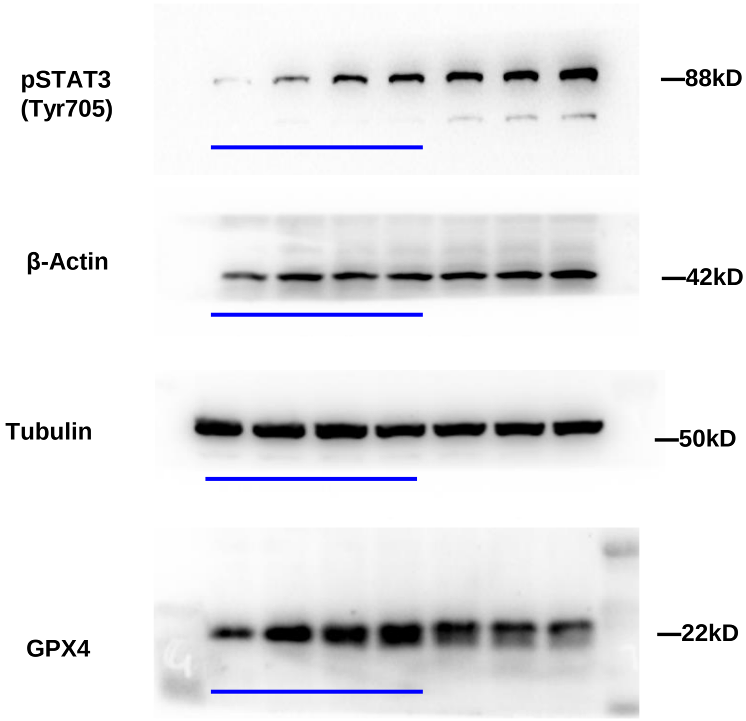

Original uncropped western blot gels

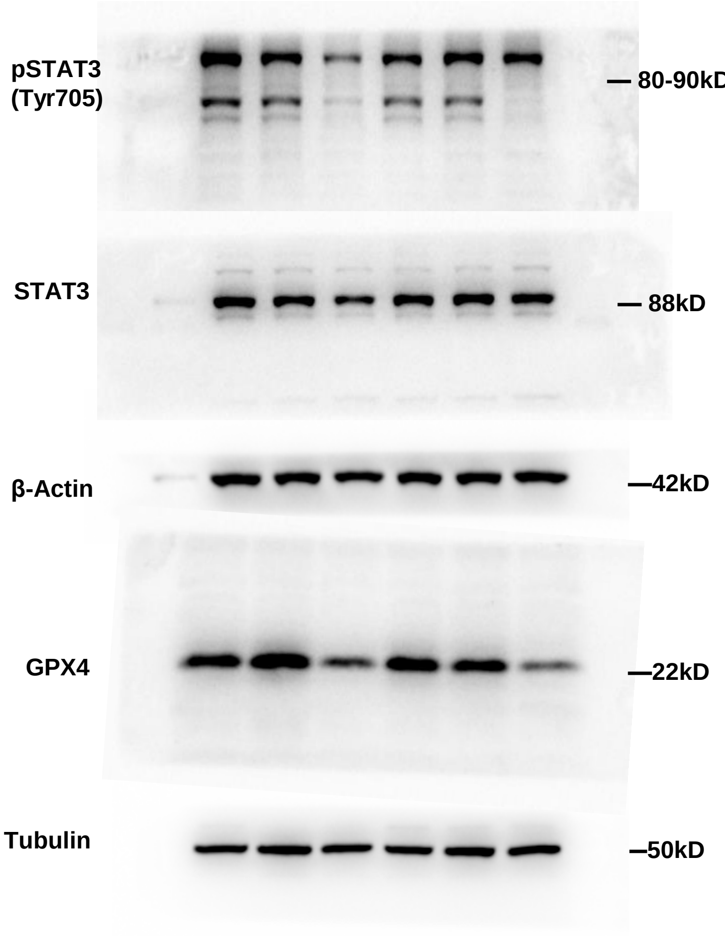

The original uncropped gels for western blots in Figure S1.

Western blots in the article

Fig.S1C

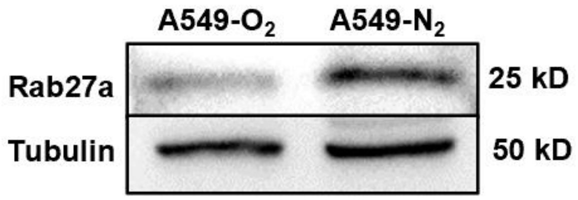

Original uncropped western blot gels

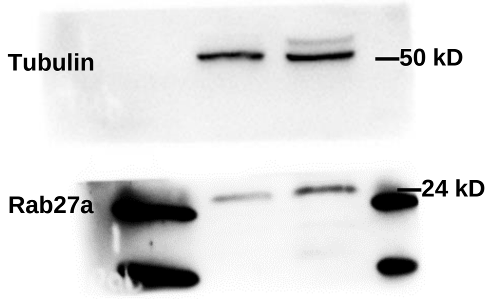

Western blots in the article

Fig.S1D

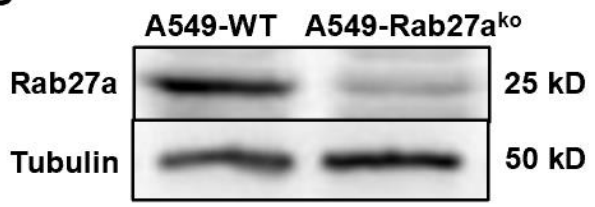

Original uncropped western blot gels

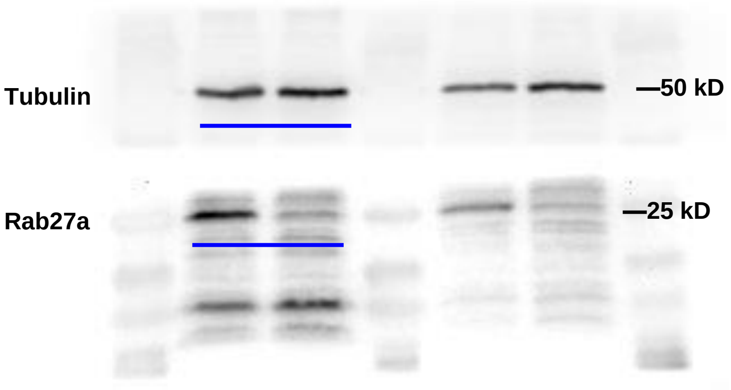

The original uncropped gels for western blots in Figure S2.

Western blots in the article

Fig. S2A

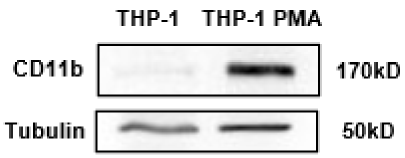

Original uncropped western blot gels

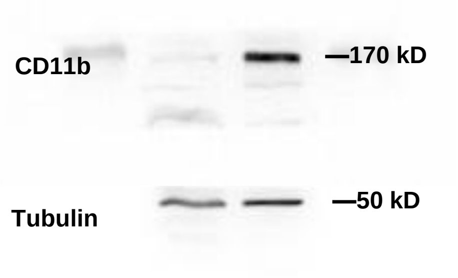

The original uncropped gels for western blots in Figure S4.

Western blots in the article

Fig.S4A

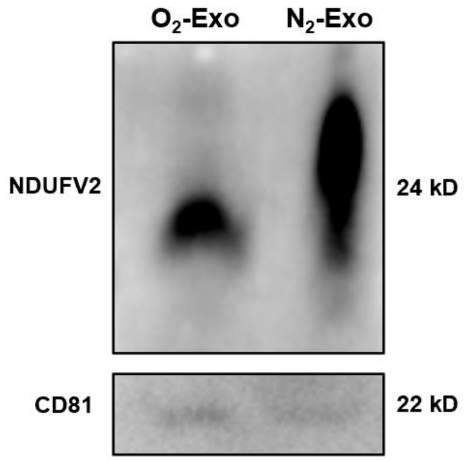

Original uncropped western blot gels

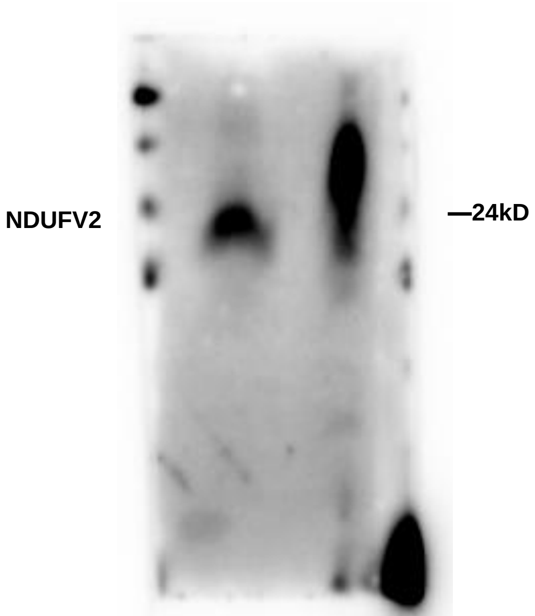

Marker on both sides led to overexposure, and the marker was covered with tin foil to show the real exposure of strip.

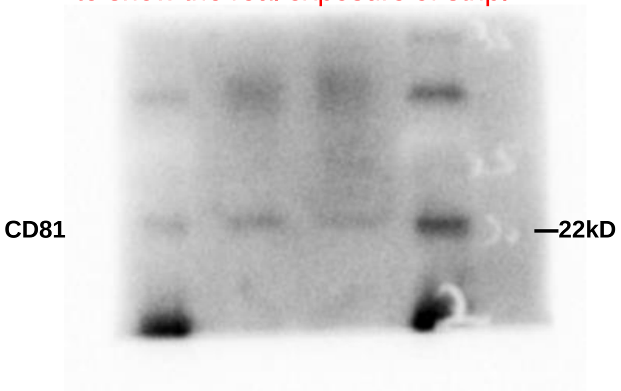

Western blots in the article

Fig.S4F

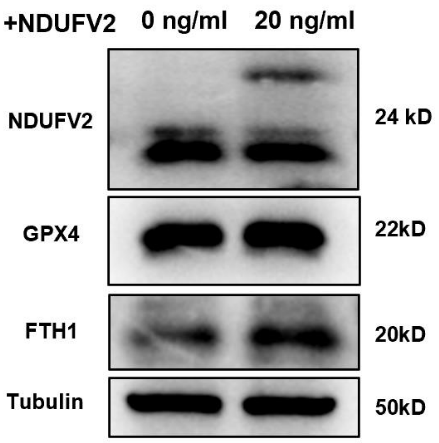

Original uncropped western blot gels

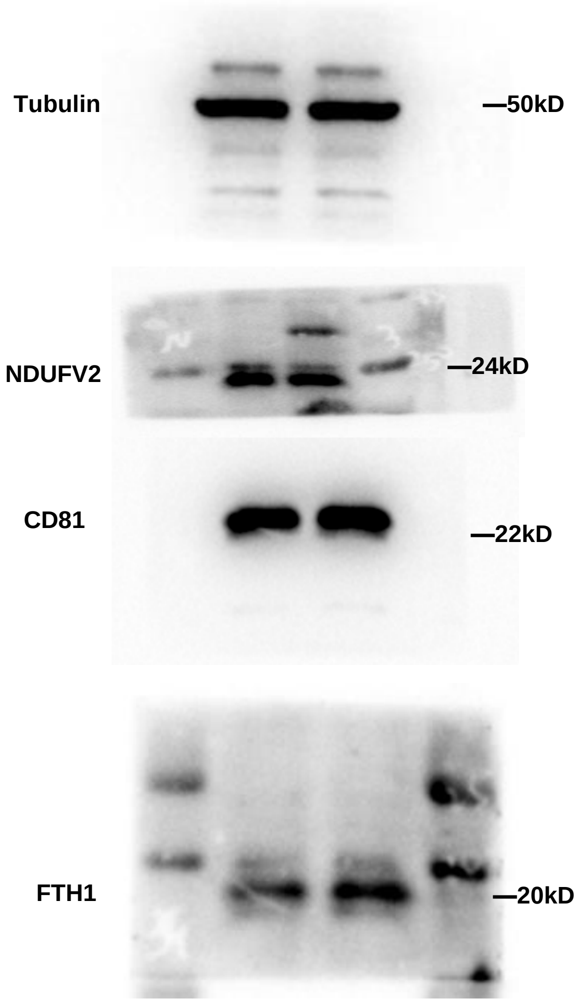

The original uncropped gels for western blots in Figure S6.

Western blots in the article

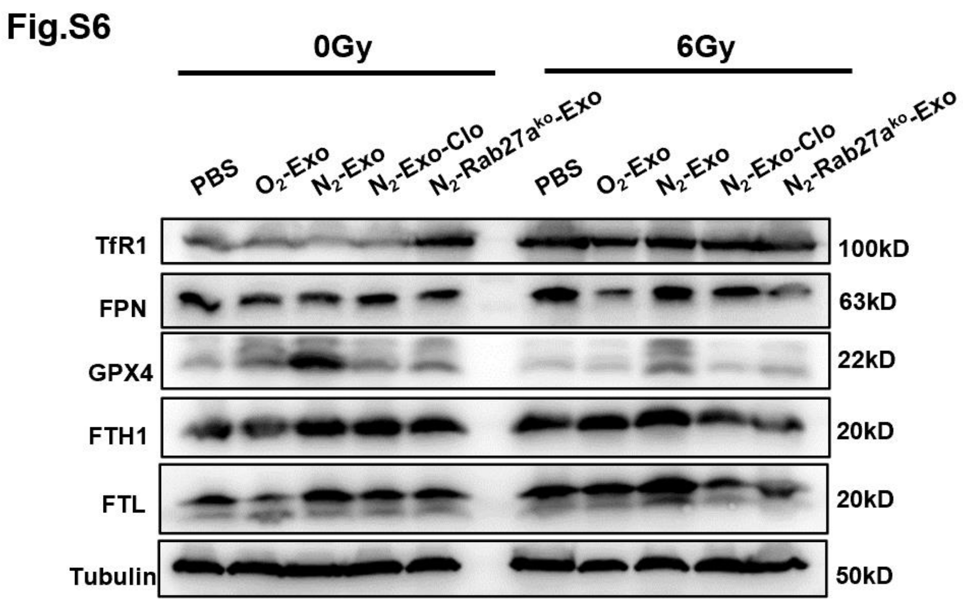

Original uncropped western blot gels

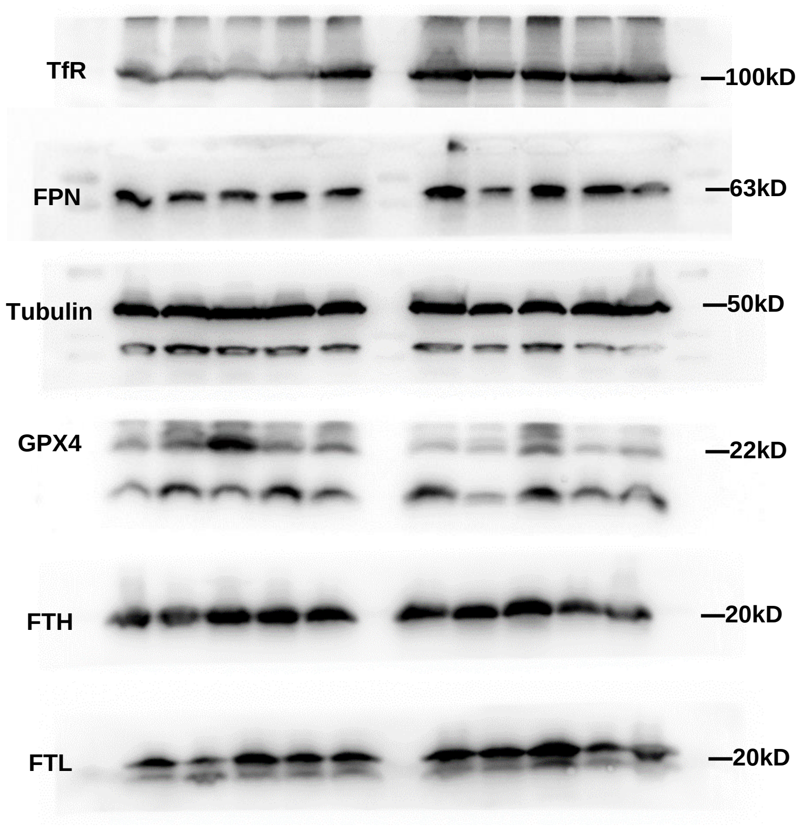

The original uncropped gels for western blots in Figure S8.

Western blots in the article

Fig. S8E

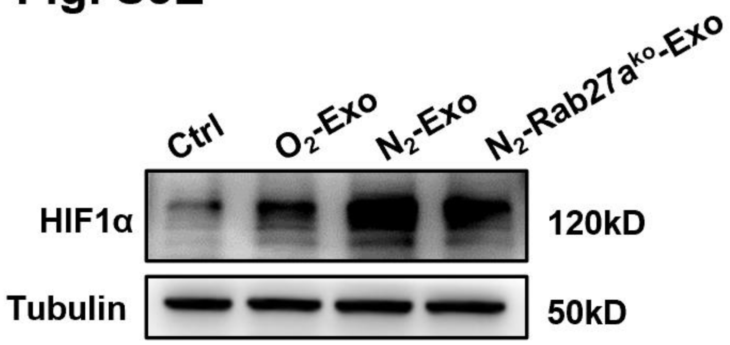

Original uncropped western blot gels

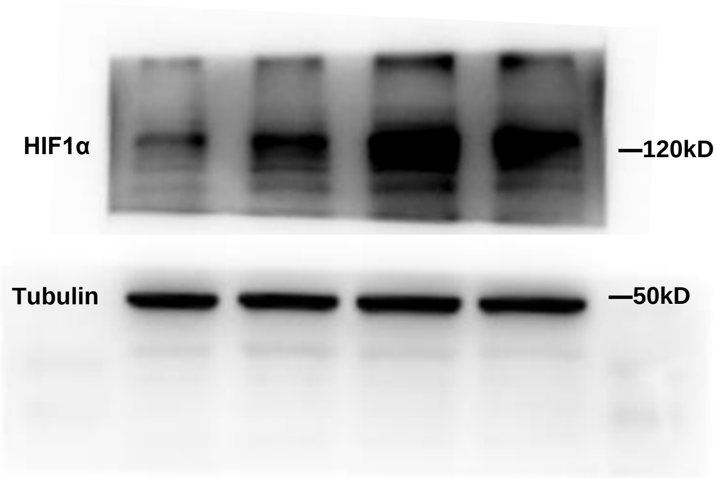

Marker on both sides led to overexposure, and the marker was covered with tin foil to show the real exposure of strip.
